# Environmental enrichment shapes striatal spike-timing-dependent plasticity *in vivo*

**DOI:** 10.1038/s41598-019-55842-z

**Published:** 2019-12-19

**Authors:** Teresa Morera-Herreras, Yves Gioanni, Sylvie Perez, Gaetan Vignoud, Laurent Venance

**Affiliations:** 1 Team Dynamic and Pathophysiology of Neuronal Networks, Center for Interdisciplinary Research in Biology, College de France, CNRS UMR7241/INSERM U1050, MemoLife Labex, Paris, France; 2 Department of Pharmacology, Faculty of Medicine and Nursing, University of the Basque Country (UPV/EHU), 48940 Leioa, Bizkaia Spain; 3 Neurodegenerative Diseases Group, BioCruces Bizkaia Health Research Institute, 48903 Barakaldo, Bizkaia Spain

**Keywords:** Neural circuits, Spike-timing-dependent plasticity

## Abstract

Behavioural experience, such as environmental enrichment (EE), induces long-term effects on learning and memory. Learning can be assessed with the Hebbian paradigm, such as spike-timing-dependent plasticity (STDP), which relies on the timing of neuronal activity on either side of the synapse. Although EE is known to control neuronal excitability and consequently spike timing, whether EE shapes STDP remains unknown. Here, using *in vivo* long-duration intracellular recordings at the corticostriatal synapses we show that EE promotes asymmetric anti-Hebbian STDP, i.e. spike-timing-dependent-potentiation (tLTP) for post-pre pairings and spike-timing-dependent-depression (tLTD) for pre-post pairings, whereas animals grown in standard housing show mainly tLTD and a high failure rate of plasticity. Indeed, in adult rats grown in standard conditions, we observed unidirectional plasticity (mainly symmetric anti-Hebbian tLTD) within a large temporal window (~200 ms). However, rats grown for two months in EE displayed a bidirectional STDP (tLTP and tLTD depending on spike timing) in a more restricted temporal window (~100 ms) with low failure rate of plasticity. We also found that the effects of EE on STDP characteristics are influenced by the anaesthesia status: the deeper the anaesthesia, the higher the absence of plasticity. These findings establish a central role for EE and the anaesthetic regime in shaping *in vivo*, a synaptic Hebbian learning rule such as STDP.

## Introduction

Behavioural experience, such as environmental enrichment (EE), has profound long-term effects on learning and memory performance^1–5^. EE, which combines complex inanimate and social interactions^6,7^, provides sensory, motor and cognitive stimulation that are normally lacking in standard rodent housing. Rearing animals in EE aims at mimicking the circumstances of a stimulating and interesting living environment that is conductive to cognitive functions. Cognitive functions are mainly characterised by synaptic plasticity that scales in both directions of the synaptic weight, widely observed with long-term potentiation (LTP) and long-term depression (LTD)^8,9^. Synaptic strength between neurons can be modified by their relative activity on either side of the synapse as postulated many decades ago by Donald Hebb^10^. More precisely, the firing of the first neuron would influence long-term the firing of the second one as a means of storing a memory trace. The corresponding cellular paradigm consists the spike-timing dependent plasticity (STDP), a synaptic Hebbian learning rule in which occurrence of timing-dependent-LTP (tLTP) or -LTD (tLTD) relies on the precise (milliseconds) relative timing of pre- and postsynaptic action potentials^11^. STDP has emerged as a candidate mechanism for experience-dependent changes in the neural circuit, including map plasticity^11^. The temporal rules of STDP are determined by the spatial location of dendritic inputs^12–14^, factors affecting dendritic voltage-gated channel properties^15^, gamma-aminobutyric acid (GABA)^16–18^, or by neuromodulators^19,20^. In brief, events modulating the electrotonic properties of the dendritic tree and thus of the back-propagating action potential, are expected to shape STDP. Although EE is known to control neuronal excitability through dendritic morphology^21^, channel, and receptor expression or modification of the balance between excitation and inhibition^1^, whether EE controls STDP remains unknown. To date, the vast majority of studies have characterised in detail the molecular, structural, and synaptic changes in animals exposed to EE by using *ex vivo* brain slices and assessed changes in synaptic plasticity using frequency-based protocols (high- or low stimulation frequency protocols). The effect of EE on synaptic strength in *ex vivo* preparations is controversial, describing either an increase in synaptic strength or an absence of an effect^22^. These discrepancies could be due to slice preparation methodology, which could alter or mask the *in vivo* EE effect in adult animals^22,23^. Thus, it is necessary to examine Hebbian synaptic plasticity *in vivo* at the single-cell level in adult rodents raised in EE.

EE is known to improve learning of procedural tasks such as the accelerating rotarod treadmill^24^. Striatal synaptic plasticity provides a fundamental mechanism for basal ganglia function for action control and procedural learning^25–30^. Here, we aimed at examining the effects of EE on STDP temporal rules in the dorsolateral striatum *in vivo*. For this purpose, we performed *in vivo* long-duration intracellular recordings (sharp pipette electrode) of striatal projecting neurons (the medium-sized spiny neurons, MSNs) to investigate STDP in rats grown in standard environment (SE) or EE with various anaesthetic regimes (deep *vs* light levels of anaesthesia). We found that EE (together with the anaesthetic level) has a crucial impact on STDP. Rats reared in SE under deep anaesthesia, displayed mainly tLTD in a wide temporal window (Δt_STDP_) (−100 < Δt_STDP_ < +100 ms) for post-pre and pre-post cortico-striatal pairings, together with a high rate of failure of plasticity expression. In contrast, rats reared in EE for two months exhibited bidirectional STDP with tLTP occurring after post-pre pairings with −100 < Δt_STDP_ < −50 ms, whereas tLTD occurred in a large temporal window, i.e. −50 < Δt_STDP_ < +100 ms. Under light anaesthesia, we observed bidirectional STDP in rats raised either in SE or EE. Interestingly, rats grown in EE displayed bidirectional STDP in a restricted temporal window, comparable to STDP observed in *ex vivo* studies, i.e. −40 < Δt_STDP_ < +40 ms, also showing a high rate of plasticity expression. In conclusion, EE promotes bidirectional STDP in a restricted temporal window with high rate of plasticity expression.

## Materials and Methods

### Animals

Experiments were conducted in adult (P_65-90_) male Sprague-Dawley rats (Charles River, L’Arbresle, France). All experimental protocols and methods (maintenance, surgery and all experiments) were approved by the local animal welfare committee (Center for Interdisciplinary Research in Biology and College de France) and by the Ethics Committee in Charge of Animal Experimentation (Paris Centre et Sud), and EU guidelines (directive on “The Protection of Animals Used for Scientific Purposes”, 2010/63/EU). Every effort was made to minimize animal suffering and to use the minimum number of animals per group and experiment. Animals were placed in SE or EE (detailed below) under controlled conditions (22 ± 1 °C, 55 ± 5% relative humidity, and 12:12 h light/dark cycle) with food and water provided *ad libitum*.

### Rearing environments

Animals were randomly distributed in two groups: (1) SE or (2) EE for eight weeks. All animals were housed in a 12 h light/dark cycle and litter, food and water were available *ad libitum*.

#### Standard housing environment

Cages for SE were standard laboratory cages (47 × 35 × 21 cm) that contained only bedding without complex inanimate and social stimulation.

#### Enriched housing environment

Based on the standard definition of EE housing (a combination of complex inanimate and social interactions^2,6,7,31^), three male rats from the same litter (from 50–60 g, i.e. 22–27 days) were housed for eight weeks in large cages (80 × 57 × 21 cm) with a running wheel, to promote physical exercise at will, and at least 3–5 differently shaped objects (tunnels, shelters, stairs, boxes, movable balls, plastic toys, nesting materials). Objects were replaced every two days during the eight weeks to ensure continuous novelty and complexity. In addition to the running wheel, caution was taken to provide at least one object aiming at decreasing stress (tunnel or shelters with various shapes and textures with increasing size and diameter according to rodent development) and another object promoting physical activity at will and exploration (toys, balls, tanks of hidden food). We used toys and balls that displayed various shapes/diameters, textures, or colours. Running wheels were randomly removed from the cage for one day every 10 days to promote novelty. Rats were exposed continuously to EE for eight weeks before evaluation of synaptic plasticity.

### *In vivo* single-unit intracellular recordings

#### Animal preparation

Animals (weighting 275–300 g, P_80-90_) were initially anaesthetized using chloral hydrate (420 mg/kg, i.p.) (Sigma-Aldrich, Saint-Quentin Fallavier, France) and placed in a stereotaxic frame (Unimecanique, Asniere, France) with their heads secured horizontally. The skull was exposed, and two 3-mm burr holes were drilled over the right dorsal striatum and the ipsilateral somatosensory cortex. Rats were continuously monitored using the electrocardiogram to ascertain that vital signs were within physiological range. Body temperature was maintained at 36.5 °C for the entire experiment with a homeothermic blanket. Anaesthesia maintenance was ensured by continuous infusion (i.p.), on demand, of chloral hydrate using a peristaltic pump.

#### Deep anaesthesia experiments

To obtain a deep level of anaesthesia, continuous infusion of chloral hydrate was administered at 60 mg/kg/h (turned on one hour after induction). Proper depth of anaesthesia was assessed regularly by testing the heart rate, spontaneous electrocorticogram (ECoG) activity (slow oscillatory pattern), the lack of response of mild hind paw pinch, and the lack of vibrissae movement. Spontaneous ECoG activity was recorded using a low impedance (~60 kΩ) silver electrode placed on the dura above the somatosensory cortex and a reference electrode inserted in a muscle at the opposite side of the head. Surface cortical signals were amplified by a differential AC amplifier (Model 1700; A-M Systems), filtered at 10 kHz (CED Micro 1401, Cambridge Electronic Design).

#### Light anaesthesia experiments

To ensure a light level of anaesthesia, continuous infusion of chloral hydrate was set at 30–40 mg/kg/h and animals were placed in painless contention. For this purpose, after surgery, the head was maintained by a metal bar rigidly fixed to the stereotaxic frame and anchored on the skull with dental cement; the ear bars and mouth piece were then removed. In this case, the suitable level of anaesthesia was adjusted to have no large delta waves with a small amplitude, fast and tonic ECoG activity.

#### *In vivo* intracellular (sharp pipette electrode) recordings

Intracellular recordings of MSNs were performed using glass microelectrodes filled with 1 M K-acetate (resistance measured in the brain between 60 and 100 MΩ) and mounted on a piezo-controlled microdrive (Burleigh Instruments, Fishers, NY, USA). Recordings were performed in the dorsal striatum, at the same anteriority as the cortical stimulation, in a region directly targeted by the afferents from the somatosensory motor cortex (stereotaxic coordinates: 0.5–3.5 mm posterior to bregma, 4.2–4.8 mm lateral to the midline and 3–5.5 mm ventral to the cortical surface). Once the electrode was placed, to obtain long-duration stable intracellular recordings, the craniotomy was filled with low melting point paraffin and the cistern was drained.

**Figure 1:**
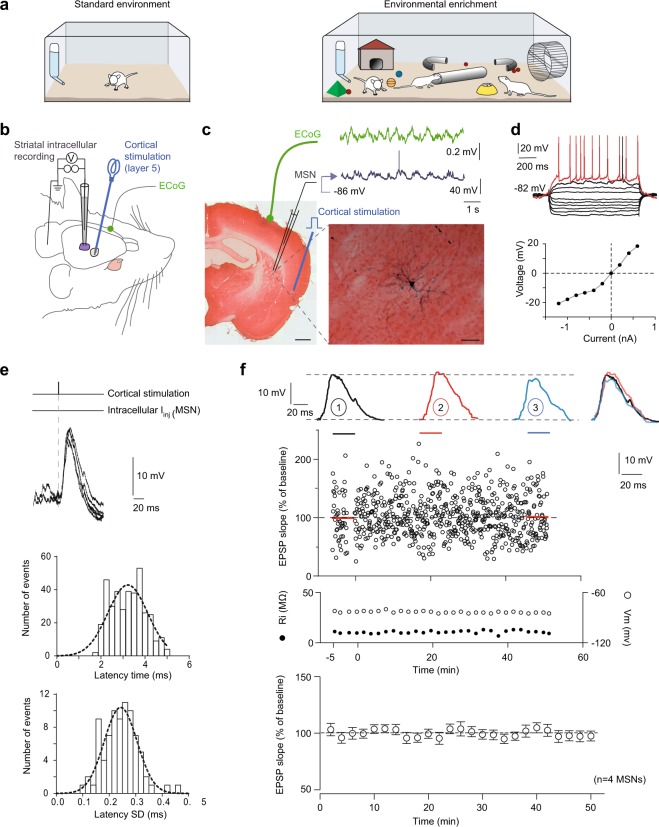
*In vivo* corticostriatal long-duration transmission at MSN. (**a**,**b**) Schematic representations of (**a**) SE and EE conditions and (**b**) of the *in vivo* experimental set-up for single-unit intracellular recording of MSNs (in the dorsolateral striatum), electrical stimulation of the ipsilateral somatosensory cortex (layer 5) and electrocorticogram (ECoG). (**c**) *In vivo* spontaneous activity of the morphologically identified biocytin-filled MSN (scale bar: 50 µm) and the simultaneous ECoG recording. Locations of the cortical stimulation and striatal recording are indicated on the brain coronal section (scale bar: 1000 µm). (**d**) Characteristic MSN voltage responses to series of 1 sec current pulses (increasing step of 0.1 nA from −0.6 to 0.6 nA) and spiking pattern of MSN. Note the hyperpolarized resting membrane potential (−82 mV), the inward rectification for hyperpolarizing current pulses (illustrated in the I/V curve) and the long latency of spike discharge for threshold current pulse (black trace). (**e**) Top, superimposed EPSPs evoked by single cortical layer 5 stimuli delivered at 0.2 Hz; bottom, latency distribution of EPSPs recorded in 82 MSNs was centred around 3.2 ms and well fitted by a Gaussian curve; SD distribution of average EPSP latency recorded in 82 MSNs was centred around 0.25 ms and well fitted by a Gaussian curve; such narrow distribution indicates a monosynaptic corticostriatal transmission. (**f**) Control experiment showing the time courses of EPSP slopes and their stability during 50 min recording by applying 0.2 Hz cortical stimulations. Upper panels: representative experiment; note that no significant variation of the EPSP slope was observed along time (1: basal EPSP slope, 2: after 20 min and 3: after 50 min); Single EPSP slopes (empty symbols) are represented. Top, average of 60 EPSP slopes before application of the protocol (0.99 ± 0.11 mV/ms; black traces), at 20 min after protocol application (0.99 ± 0.08 mV/ms; red traces) and at 50 min after protocol application (0.95 ± 0.06 mV/ms; blue traces). The stable time courses of Ri and Vm are illustrated for this cell; for clarity, average of 15 Ri and Vm are represented. Bottom, summary of experiments (n = 4 MSNs) showing the average and normalised time courses of EPSP slopes and their stability during a 50 min recording.

All recordings were obtained using an Axoclamp-2B amplifier (Molecular Devices, San Jose, CA, USA) configured in current-clamp mode and the bridge was balanced manually online (−0.3 nA, 50 ms current pulses). Impalements of neurons were considered successful when the membrane potential (Vm) was at least −60 mV and spike amplitude >50 mV. Current-clamp recordings were filtered at 10 kHz. Electrical activity was acquired and directly sampled at 10 kHz with a CED Micro1401 (Cambridge Electronic Design, Cambridge, UK) and analysed *off-line* with the Spike2 software (Cambridge Electronic Design, Cambridge, UK). MSNs were distinguished from interneurons based on passive and active membrane properties^32^ (see Fig. 1). Ri of neurons was assessed with the mean steady-state voltage deflections during hyperpolarizing current pulses (−0.3 nA, 50 ms duration, every 5 s) applied intracellularly through the recording electrode. Current-voltage relationships were obtained by injecting hyperpolarizing and depolarizing current pulses through the microelectrode (increasing step of 0.1 nA from −0.6 to 0.6 nA). Subsequently, some of the recorded neurons were identified morphologically (see Methods below).

#### STDP induction protocols

Electrical stimulations of the cerebral cortex were performed with a concentric bipolar electrode (300 µm diameter, 300 µm tip-barrel distance) (Phymep, Paris, France) placed in layer 5 of the somatosensory cortex (stereotaxic coordinates: −1.8 mm to bregma, 5.8–6 mm to the midline and 3.5 mm ventral to the dura mater). Electrical stimulations (100 µs) were monophasic at constant current (Pulsemaster A300, WPI, Stevenage, UK). Current pulse intensity (0.1–1 mA) was adjusted to evoke striatal subthreshold excitatory post-synaptic potentials (EPSPs) ranging from 5 to 20 mV. Repetitive control stimuli were applied at 0.2 Hz. At the end of each *in vivo* recording session, we verified the cortical localization of the stimulating electrode. The position of the electrode was marked by electrolytic lesion by passing a current of ±50 mA during 1 s, which was then observed on histological sections (50 µm) after safranine staining. The control baseline recording consisted of at least 50–60 responses to cortical stimuli delivered at 0.2 Hz. STDP induction protocols of pre- and post-synaptic stimulation pairings (100–120 times at 1 Hz) with a time shifting (Δt_STDP_) of several milliseconds, corresponding to the duration between the stimulation artefact and the peak of the post-synaptic spike. Pre-synaptic stimulations corresponded to cortical stimulations and post-synaptic stimulations to a depolarizing step current (30 ms duration) intracellularly injected through the recording electrode (the intensity of this current step, 0.2–29 nA, was adjusted to evoke 1–3 action potentials in the MSN). We chose somatic current injection to evoke post-synaptic action potentials because somatosensory stimulations (vibrissae) hardly evoke MSN firing under anaesthesia^33^ (data not shown). After STDP pairings, MSNs were recorded with 0.2 Hz cortical stimuli for 30–60 minutes. Long-term synaptic efficacy changes were measured after 50 min. Input resistance (assessed with hyperpolarizing current of −0.3 nA for 50 ms) and Vm were monitored throughout the experiment (both measured every 5 sec) and variation >20% led to exclusion of the experiment.

#### Electrophysiological data analysis

**Figure 2:**
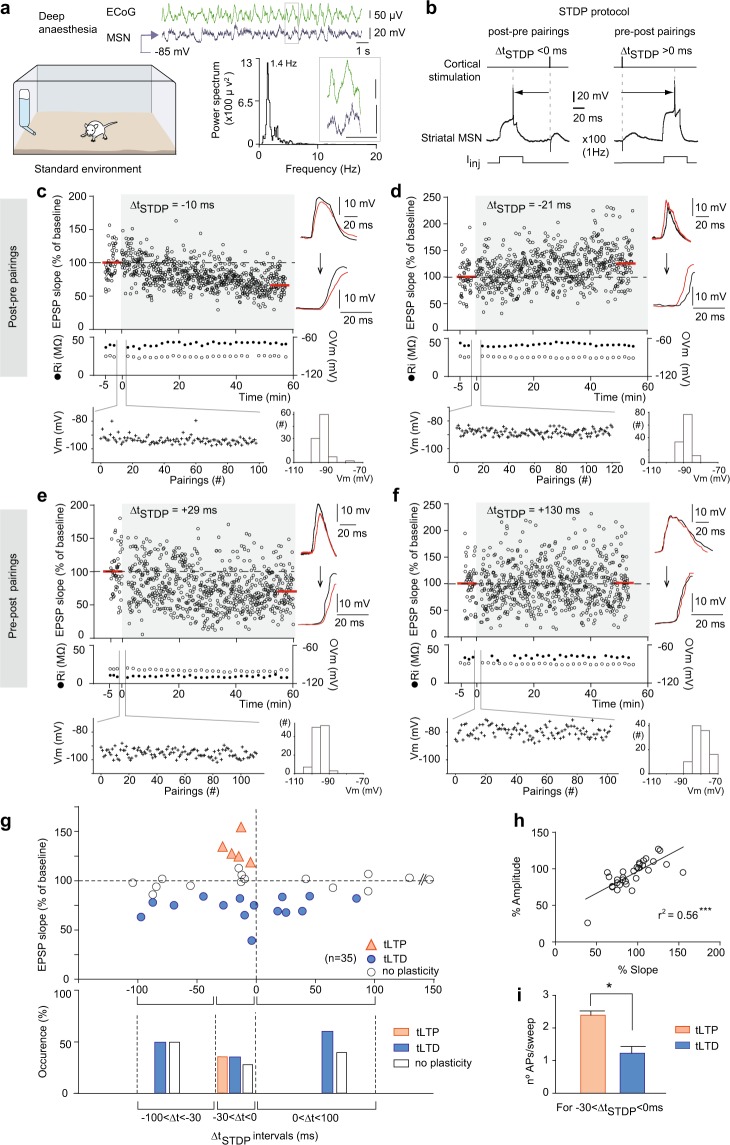
*In vivo* corticostriatal STDP in deeply anaesthetised rats raised in standard housing. (**a**) Top, intracellular recording of the spontaneous activity of a single MSN (Vm = −85 mV) together with the corresponding ECoG waves showing the characteristic up and down states observed in deeply anaesthetised rats; bottom, spectral analysis of the ECoG recordings showing a strong periodicity at 1.4 Hz corresponding to a deep level of anaesthesia. (**b**) STDP induction protocol: an action potential evoked in one MSN was paired a few milliseconds before (post-pre pairings) (left side) or after (pre-post pairings) (right side) with a cortical stimulation; this pairing was repeated 100 times at 1 Hz. ∆t_STDP_ indicates the time shift between pre- and post-synaptic stimulations. ∆t_STDP_ < 0 ms and ∆t_STDP_ > 0 ms refer to post-pre and pre-post pairings, respectively. (**c**–**f**) Representative experiments illustrating the time courses of synaptic efficacy changes induced by STDP protocols. Bottom panels: evolution of input resistance (Ri) (●) and Vm (○) illustrates the stability of the recordings during the whole experiment (averaged every 2 min) and the Vm during STDP pairings (Vm just before each pairing as a function of the number of pairings). Representative traces are the average of 60 EPSPs during baseline (black traces) and 55–60 min after STDP pairings (red traces). (**c**) Example of tLTD induced by 100 post-pre pairings with Δt_STDP_ = −10 ms. Top, EPSP slope before and after 100 pairings (before pairings: 2.00 ± 0.06 mV/ms; 55–60 min after pairings: 1.32 ± 0.05 mV/ms; decrease of 65%). Bottom, time courses of Ri (before, 38.7 ± 0.9 MΩ; after, 40.7 ± 0.3 MΩ; change of 5%) and Vm (before, −90.0 ± 0.6 mV; after, −90.0 ± 0.6 mV; no change) for this cell. (**d**) Example of tLTP induced by 100 post-pre pairings with Δt_STDP_ = −21 ms. Top, EPSP slope before and after 100 pairings (before pairings: 0.87 ± 0.04 mV/ms; 55–60 min after pairings: 1.11 ± 0.07 mV/ms; increase of 128%). Bottom, time courses of Ri (before, 41.7 ± 1.2 MΩ; after, 40.3 ± 0.3 MΩ; change of 3%) and Vm (before, −89.0 ± 0.6 mV; after, −90.3 ± 0.7 mV; change of 1%) for this cell. (**e**) Example of tLTD induced by 100 pre-post pairings with Δt_STDP_ = +29 ms. Top, EPSP slope before and after 100 pairings (before pairings: 2.47 ± 0.12 mV/ms; 55–60 min after pairings: 1.71 ± 0.15 mV/ms; decrease of 69%). Bottom, time courses of Ri (before, 10.3 ± 0.7 MΩ; after, 8.3 ± 0.3 MΩ; change of 19%) and Vm (before, −97.3 ± 0.3 mV; after, −98.3 ± 0.3 mV; change of 1%) for this cell. (**f**) Example of the absence of plasticity induced by 100 pre-post pairings with Δt_STDP_ = +130 ms. Top, EPSP slope before and after 100 pairings (before pairings: 0.95 ± 0.55 mV/ms; 55–60 min after pairings: 0.98 ± 0.46 mV/ms; variation of 3%). Bottom, time courses of Ri (before, 34.0 ± 1.5 MΩ; after, 34.0 ± 1.7 MΩ; change of 0%) and Vm (before, −83.0 ± 0.6 mV; after, −84.3 ± 0.3 mV; change of 2%) for this cell. (**g**) Summary of STDP experiments (n = 35) showing a dominance of tLTD for post-pre and pre-post pairings (−100 < Δt_STDP_ < +100 ms) with bidirectional plasticity expression for −30 < Δt_STDP_ < 0 ms. Each symbol represents one neuron in which synaptic efficacy estimated at least 50 min after STDP induction. Bottom, summary bar graph illustrating the occurrence of tLTP, tLTD or no plasticity. Note the important failure rate for plasticity induction and tLTP expression exclusively for post-pre pairings in narrow Δt_STDP_ (−30 < Δt_STDP_ < 0 ms). The point located after the interruption of the x-axis is at Δt_STDP_ = +208 ms. (**h**) Significant correlation between EPSP amplitude and slope changes after STDP induction protocols (*p* < 0.0001, linear regression analysis). (**i**) Significant relationship between the number of postsynaptic action potentials evoked during the post-pre pairings and the polarity of plasticity (−30 < Δt_STDP_ < 0 ms) (*p* = 0.0323, unpaired Student *t*-test).

Off-line analysis was performed using the Spike2 software (Cambridge Electronic Design, Cambridge, UK) and Igor-Pro 6.0.3 (Wavemetrics, Lake Oswego, OR, USA). The strength of the synaptic transmission was measured as the EPSP slope. A linear fit to the ascending phase of the EPSP was calculated from a time corresponding to 10% of the EPSP amplitude to the top. Throughout the recording, every two minutes, 24 successive EPSPs were individually measured and normalized to the mean of the baseline. The 24 normalized EPSP slopes were then averaged and expressed as mean ± SEM. Synaptic efficacy changes measured after 50 min of the STDP induction protocol were classified as either tLTP or tLTD when the mean normalized EPSP slope (corresponding to the last 4–5 min of the recording) was significantly different from the control baseline. For each cell, the value reported on the graphs represents the mean of the last 50–60 EPSP slope measurements. Note that, in parallel, the amplitude of EPSPs (from the basal Vm to the EPSP peak) and the EPSP area (integral) were also measured. In all cases, results (tLTP, tLTD and absence of plasticity) were similar with the estimated amplitude, area and slope of EPSPs (see Fig. 2h for correlation between EPSP amplitude and slope). When considered altogether (n = 85 neurons), correlations between EPSP slopes and either peak amplitudes or areas were significant: r^2^ = 0.55 (*p* < 0.0001) and r^2^ = 0.33 (*p* < 0.0001), respectively.

Spectral analysis of ECoG potentials was performed by applying Fast Fourier Transforms using Spike2 (Cambridge Electronic Design, Cambridge, UK).

Statistical analysis was performed using the Prism 5.0 software (San Diego, CA, USA). All results were expressed as mean ± SEM. Statistical significance was assessed using the Student’s unpaired/paired *t*-tests or one-way ANOVA for variables normally distributed (Vm, Vm SD, VM UP and Down states, firing threshold, action potential amplitude and number of action potentials per pulse). However, to assess differences in variables with non-normal distribution (Ri, rheobase, EPSP amplitude, amplitude of LTP/LTP/no plasticity and %EPSPS slope *vs* basal), Wilcoxon matched-pairs signed rank test or Kruskal-Wallis test were used. The Kolmogorov-Smirnov test was used for assessment of normality. Linear regression analysis was conducted for correlations (SigmaStat version 3.5, Systat Software). The level of statistical significance was set at *p* < 0.05.

We measured the mean Vm before each stimulation during the baseline, during and after STDP pairings. To do so, we detected stimulations using two thresholds (one for the potential and one for its derivative). When a stimulation was detected, we measured the mean potential values over 20 ms (starting from 1 ms preceding stimulation). Then, we computed for each experiment the mean and variance of Vm before stimulations, in the three different phases. For post-STDP pairings, potentials were measured at the end of the recordings over five minutes. The algorithm was implemented in Python (2.7 and 3.6), using the anaconda suite (Anaconda Software Distribution, Computer software Version 2–2.4.0. Nov 2016, Web. https://anaconda.com).

Spearman correlation tests were conducted between the mean/variance of Vm during the three experimental phases and the relative synaptic efficacy changes. Pearson correlations were also computed for Vm in the five minutes baseline preceding STDP pairings and in the last five minutes of the recordings (post-STDP pairings). We report the *p*-values in Supplementary Figs. 1–4. Statistical analysis was performed using the scipy.stats Python library.

### Whole-cell patch-clamp recordings in acute brain slices

#### Brain slice preparation

Horizontal brain slices (thickness 330 µm) from adult (P65-90) Sprague-Dawley male rats (Charles River), preserving corticostriatal connections between somatosensory cortex layer 5 and dorsolateral striatum^34^, were prepared as previously described^17^ by using a vibrating blade microtome (VT1200S, Leica Microsystems, Nussloch, Germany). Brains were sliced in an ice-cold cutting solution (125 mM NaCl, 2.5 mM KCl, 25 mM glucose, 25 mM NaHCO_3_, 1.25 mM NaH_2_PO_4_, 2 mM CaCl_2_, 1 mM MgCl_2_, 1 mM pyruvic acid) bubbled with 95% O_2_/5% CO_2_.

#### Patch-clamp electrophysiological recordings

Patch-clamp recordings were performed as previously described^16,17,34^. Briefly, borosilicate glass pipettes with 4–6 MΩ resistance were used for whole-cell recordings (in mM): 122 K-gluconate, 13 KCl, 10 HEPES, 10 phosphocreatine, 4 ATP-Mg, 0.3 GTP-Na, 0.3 EGTA (adjusted to pH 7.35 with KOH). The composition of the extracellular solution was (mM): 125 NaCl, 2.5 KCl, 25 glucose, 25 NaHCO_3_, 1.25 NaH_2_PO_4_, 2 CaCl_2_, 1 MgCl_2_, 10 μM pyruvic acid bubbled with 95% O_2_ and 5% CO_2_. Signals were amplified using an EPC10-2 amplifier (HEKA Elektronik, Lambrecht, Germany). All recordings were performed at 34 °C using a temperature control system (Bath-controller V, Luigs & Neumann, Ratingen, Germany) and slices were continuously superfused at 2–3 ml/min with the extracellular solution. Current- and voltage-clamp recordings were sampled at 10 kHz, with the Patchmaster v2x32 program (HEKA Elektronik).

#### STDP induction protocols

According to previous studies^16,17,34^, electrical stimulations were performed with a concentric bipolar electrode (Phymep, Paris, France) placed in layer 5 of the somatosensory cortex and were monophasic at constant current (ISO-Flex stimulator, AMPI, Jerusalem, Israel). Currents were adjusted to evoke 50–200 pA EPSCs. Repetitive control stimuli were applied at 0.1 Hz. STDP protocols consisted of pairings of pre- and post-synaptic stimulations (at 1 Hz) at a fixed Δt_STDP_. Pre-synaptic stimulations corresponded to cortical stimulations and the post-synaptic stimulation to an action potential evoked by a depolarizing current step (30 ms duration) in MSNs. MSNs were recorded for 10 min during baseline and for at least 60 min after STDP protocol; long-term synaptic efficacy changes were measured after 50 min. The amplitudes of 30 successive EPSCs were individually measured and the average was calculated. Variation of input and series resistance (Ri and Rs) above 20% led to exclusion of the experiment.

#### Trichloroethanol treatment

After recording a baseline in control conditions (10 min), trichloroethanol (5 mM, directly diluted in the extracellular solution) was bath-applied. After 10 min (required time to allow for total perfusion of the drug), a baseline with trichloroethanol was recorded for 10 min before application of the STDP protocol. Trichloroethanol was continuously applied until the end of the experiment.

#### Patch-clamp data analysis

Analysis was performed *off-line* using Fitmaster (Heka Elektronik) and Igor-Pro 6.0.3 (Wavemetrics, Lake Oswego, OR, USA). All results were expressed as the mean ± S.E.M. and “n” refers to a single-cell experiment from a single slice. Statistical analysis was performed with the Prism 5.0 software (GraphPad Software Inc., CA, USA) and statistical significance was assessed in Wilcoxon signed rank test using the indicated significance threshold (*p*).

### Immunohistochemistry

In a subset of experiments, intracellular glass micropipettes were filled with 1.5% biocytin (Sigma), dissolved in 2 M potassium acetate. MSNs were passively filled during at least 80 min of recordings. At the end of the electrophysiological experiment, rats were deeply anaesthetised (sodium pentobarbital, 150 mg⁄kg i.p.) and transcardially perfused with saline followed by 4% ice-cold paraformaldehyde in 0.1 M phosphate buffer. Brains were extracted and, following 2 h in the fixative solution, were transferred to 30% sucrose. Brains were then cut in frontal 50-µm sections using a freezing microtome and slices were maintained in 0.1 M potassium-PBS (pH = 7.4). To recover the recorded cell and visualize the biocytin-filled neurons, avidin-biotin-horseradish peroxidase was used (ABC Elite peroxidase kit; Vector Laboratories, Burlingame, CA, USA) according to manufacturer’s instructions.

## Results

### Properties of corticostriatal transmission *in vivo*

To study the effect of EE on corticostriatal STDP, we recorded MSNs *in vivo* from rats grown in SE or EE with various anaesthetic regimes (Fig. 1a; see Methods). We performed *in vivo* intracellular recordings in current-clamp mode of MSNs located in the dorsolateral striatum from adult rats (P_80-90_) with stimulation in the somatosensory cortex (layer 5) (n = 85) (Fig. 1b,c). We performed sharp electrode intracellular recordings because this technique ensures limited cytoplasm washout through the recording pipette, required for the long-duration recordings. Chloral hydrate anaesthetised rats were used to obtain stable intracellular recordings along time; because of the anaesthesia, cortical inputs were electrically stimulated to evoke striatal EPSPs. The recording session started when the depth of anaesthesia (deep or light) was ensured based on the power spectrum of surface ECoG activity (see example for deep anaesthesia in Figs. 1c). MSNs were electrophysiologically identified, and distinguished from the striatal interneurons, based on passive and active membrane properties^32,35^, such as hyperpolarized resting Vm (−84 ± 0.8 mV, n = 85), low input resistance (34 ± 2 MΩ), inward rectifying I/V relationship and long-latency spike discharge at rheobase (Fig. 1d). Post-recording morphological analysis performed in a subset of experiments confirmed the identity of the recorded MSNs (n = 5) (Fig. 1c). Stimulation of the ipsilateral corticostriatal afferents evoked EPSPs in MSNs with short latency (3.2 ± 0.1 ms, n = 85) and latency SD < 1 ms (0.25 ± 0.01 ms, n = 85) indicating a monosynaptic feature of this transmission (Fig. 1e). Once the corticostriatal transmission occurred, no failure was observed in MSNs, denoting a reliable and efficient transmission.

**Figure 3:**
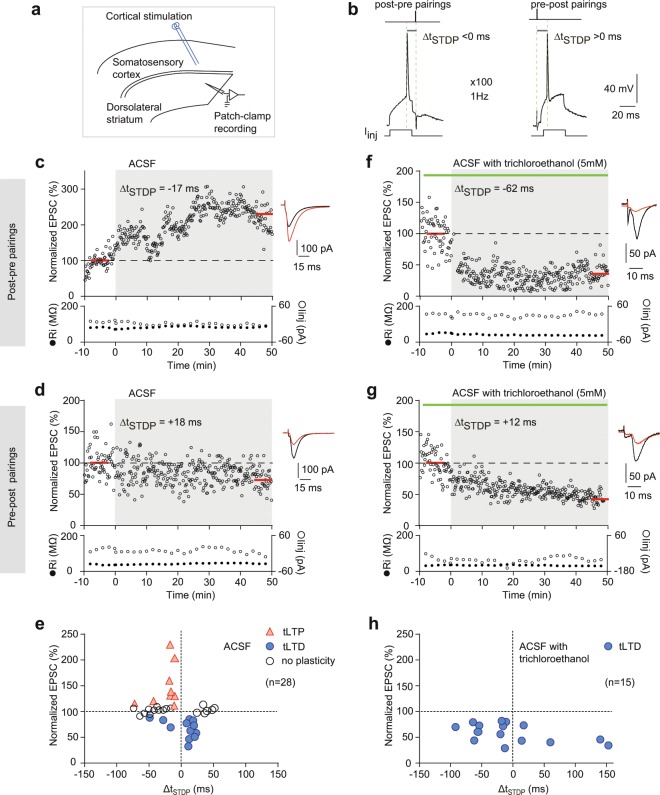
Impact of anaesthesia on *in vitro* corticostriatal STDP in adult rats. (**a**) Schematic representation of the *in vitro* experimental set-up for patch-clamp recording of MSN (in the dorsolateral striatum) and electrical stimulation of the somatosensory cortex (layer 5) in corticostriatal horizontal slices. (**b**) STDP induction protocol: an action potential evoked in a single MSN was paired a few milliseconds before (post-pre pairings) (left side) or after (pre-post pairings) (right side) with a cortical stimulation; this pairing was repeated 100 times at 1 Hz. ∆t_STDP_ indicates the time shift between pre- and post-synaptic stimulations. ∆t_STDP_ < 0 ms and ∆t_STDP_ > 0 ms refer to post-pre and pre-post pairings, respectively. (**c**–**e**) *In vitro* corticostriatal STDP in adult animals (P_65-90_). (**c**) Example of tLTP induced by 100 post-pre pairings with Δt_STDP_ = −17 ms. Top, EPSC amplitude before and after 100 pairings (before pairings: 144 ± 35 pA; 55–60 min after pairings: 331 ± 26 pA; increase of 230%). Bottom, time courses of input resistance (Ri) (●) (before, 78.4 ± 3.7 MΩ; after, 84. 5 ± 1.7 MΩ; change of 8%) and injected current (linj) (○) (before, 9.2 ± 2.2 pA; after, −5.7 ± 1.5 pA; change of 6%) for this cell. (**d**) Example of tLTD induced by 100 pre-post pairings with Δt_STDP_ = +18 ms. Top, EPSC amplitude before and after 100 pairings (before pairings: 156 ± 34 pA; 55–60 min after pairings: 113 ± 26 pA; decrease of 73%). Bottom, time courses of Ri (before, 36.4 ± 0.3 MΩ; after, 43.4 ± 0.3 MΩ; change of 18%) and linj (before, 19.5 ± 1.3 pA; after, 18.9 ± 1.8 pA; change of 3%) for this cell. (**e**) Summary of STDP experiments (n = 40) showing bidirectional plasticity expressed in a restricted time window (60 ms) and centred on Δt_STDP_ = 0 ms. (**f**–**h**) Effect of bath-applied trichloroethanol (5 mM) on *in vitro* corticostriatal STDP in adult animals (P_65-90_). (**f**) Example of tLTD induced by 100 post-pre pairings with Δt_STDP_ = −62 ms. Top, EPSC amplitude before and after 100 pairings (before pairings: 65 ± 2 pA; 55–60 min after pairings: 29 ± 3 pA; decrease of 44%). Bottom, time courses of Ri (before, 45.3 ± 2.3 MΩ; after, 37.2 ± 0.4 MΩ; change of 17%) and linj (before, 31.2 ± 3.1 pA; after, 33.3 ± 1.1 pA; change of 7%) for this cell. (**g**) Example of tLTD induced by 100 pre-post pairings with Δt_STDP_ = +12 ms. Top, EPSC amplitude before and after 100 pairings (before pairings: 93 ± 3 pA; 55–60 min after pairings: 43 ± 1 pA; decrease of 46%). Bottom, time courses of Ri (before, 36.4 ± 0.4 MΩ; after, 31.5 ± 0.3 MΩ; change of 14%) and linj (before, −112.3 ± 6.2 pA; after, −106.8 ± 3.0 pA; change of 5%) for this cell. (**h**) Summary of STDP experiments (n = 15) showing unidirectional plasticity under trichloroethanol with tLTD dominance for both post-pre or pre-post pairings and with an enlarged Δt_STDP_.

To evaluate long-term synaptic weight changes, only long-duration recordings were included in this study. First, we assessed the EPSP slope stability during 50 min recordings (by applying 0.2 Hz cortical test stimulations) in 4 MSNs. As exemplified in Fig. 1f, no significant variation in EPSP slopes was observed along time (basal EPSP slope: 100 ± 6% of the baseline; after 20 min: 108 ± 5%; after 50 min: 103 ± 5%; *p* = 0.5210); note the stable time course of Ri and Vm. Overall, as shown in the summary time course (Fig. 1f), we did not observe significant variation in EPSP slopes along time (mean value of the EPSP slope recorded after 50 min: 95 ± 2%, *p* = 0.066, n = 4; 4/4 cells showed not significant change in synaptic weight); none of the points was significantly different from the baseline average (the first five minutes) (*p* = 0.0767, Kruskal-Wallis test). We thus ensured that long-duration intracellular recordings do not affect significantly the EPSP slope, make it an appropriate technique for characterizing *in vivo* STDP.

### *In vivo* STDP in deeply anaesthetised rats grown in standard housing

We examined the influence of the temporal relationship between MSN firing and activation of corticostriatal afferents on the induction of synaptic plasticity (STDP), in adult rats grown in SE and deeply anaesthetised. Depth of anaesthesia was continuously assessed by recording spontaneous ECoG activity and was adjusted until the power spectral analysis indicated a deep level of anaesthesia (dominant frequency in the 1–3 Hz range, as exemplified in Fig. 2a). Under these conditions, recorded MSNs (n = 35) displayed an average Vm of −84 ± 1 mV, input resistance of 38 ± 3 MΩ (Table 1) and showed Vm periodic transitions between up and down states (magnitude of the fluctuations: 19 ± 0.8 mV) (Fig. 2a, Table 1 and Supplementary Fig. 1). We applied STDP pairings consisting of 100 stimulations at 1 Hz of the somatosensory cortex paired with post-synaptic suprathreshold depolarization (evoking 1–3 spikes) with a time shifting (Δt_STDP_). Δt_STDP_ < 0 refers to post-pre pairings (post-synaptic stimulation precedes pre-synaptic stimulation), whereas Δt_STDP_ > 0 refers to pre-post pairings (pre-synaptic stimulation precedes post-synaptic stimulation) (Fig. 2b).

**Table 1:** Passive and active membrane properties and characteristics of corticostriatal synaptic evoked-responses of *in vivo* recorded MSNs.

	SE & deep anaesthesia^(a)^ (n = 35)	SE & light anaesthesia^(b)^ (n = 14)	EE & deep anaesthesia^(c)^ (n = 19)	EE & light anaesthesia^(d)^ (n = 18)	Statistics^#^
*Membrane potential*					
Vm (mV)	−84 ± 1	−82 ± 1	−82 ± 2	−79 ± 1	ns
Vm SD	4.5 ± 0.2	3.4 ± 0.233	5.1 ± 0.3	3.4 ± 0.3	*p* < 0.0001 (*a *vs* d; **b *vs* c; ***c *vs* d)
Vm UP state (mV)	−71 ± 1	−73 ± 1	−70 ± 1	−71 ± 1	ns
Vm DOWN state (mV)	−90 ± 1	−88 ± 1	−90 ± 1	−86 ± 1	ns
DOWN to UP difference (mV)	19 ± 1	16 ± 1	20 ± 1	16 ± 1	*p* = 0.0005 (*a vs d, **b *vs* c; **c *vs* d)
*Cellular properties*					
Ri (Ω)	38 ± 3	25 ± 2	36 ± 3	33 ± 3	ns
Rheobase (nA)	0.47 ± 0.04	0.46 ± 0.06	0.51 ± 0.06	0.61 ± 0.12	ns
Firing threshold (mV)	−50 ± 1	−50 ± 1	−50 ± 1	−48 ± 1	ns
Action potential amplitude (mV)	59 ± 1	63 ± 1	56 ± 1	58 ± 2	*p* = 0.0446 (*b *vs* c)
Action potential duration (ms)	0.52 ± 0.01	0.39 ± 0.01	0.52 ± 0.02	0.40 ± 0.01	*p* < 0.0001 (***a *vs* b, a *vs* d, b *vs* c, c *vs* d)
*Corticostriatal synaptic responses*					
EPSP slope (mV/ms)	12.1 ± 1.2	14.2 ± 1.7	16.7 ± 3.6	15.7 ± 2.6	ns
EPSP area (mV. ms)	327 ± 27	299 ± 48	362 ± 47	281 ± 33	ns
EPSP amplitude (mV)	13.6 ± 0.8	13.8 ± 1.4	14.6 ± 1.4	14.0 ± 1.4	ns

Data are mean ± SEM; “n” refers to a single cell experiment. SE: Standard housing environment; EE: enriched housing environment; Ri: input resistance; EPSP: excitatory post-synaptic potential. ^#^Vm SD, DOWN to UP difference and action potential amplitude: one-way ANOVA (Bonferroni post-hoc test); action potential duration: Kruskal-Wallis (Dunn’s multiple comparison post-hoc test). ns: Not significant.

In adult rats grown in SE and deeply anaesthetised, STDP pairings induced mainly a dominance of tLTD within a wide Δt_STDP_ range (−100 < Δt_STDP_ < +100 ms) together with a high rate of absence of plasticity (Fig. 2).

Post-pre pairings (−100 < Δt_STDP_ < 0 ms), among 23 successful experiments, induced 9 tLTD, 9 absences of plasticity and 5 tLTP. Figure 2c shows an example of tLTD induced by 100 post-pre pairings (Δt_STDP_ = −10 ms), where the mean baseline EPSP slope was 2.00 ± 0.06 mV/ms before pairings, and decreased by 65% to 1.32 ± 0.05 mV/ms one hour after pairings; note the stable time courses of Ri and Vm. Figure 2d shows an example of tLTP induced by 100 post-pre pairings (Δt_STDP_ = −21 ms), where the mean baseline EPSP slope was 0.87 ± 0.04 mV/ms before pairings, and increased by 128% to 1.11 ± 0.07 mV/ms one hour after pairings.

With pre-post pairings, we performed 12 successful experiments, including 6 tLTD and 6 absences of plasticity. More specifically, we observed 60% of tLTD and 40% of absence of plasticity (n = 10) for 0 < Δt_STDP_ < +100 ms, and two absences of plasticity for Δt_STDP_ > +100 ms. Figure 2e shows an example of tLTD induced by 100 pre-post pairings (Δt_STDP_ = +29 ms), where the mean baseline EPSP slope was 2.47 ± 0.12 mV/ms before pairings and decreased by 69% to 1.71 ± 0.15 mV/ms one hour after pairings. A total of 100 pre-post pairings with large Δt_STDP_ (Δt_STDP_ > +100 ms, n = 2) did not induce any plasticity, as shown in Fig. 2f (Δt_STDP_ = +130 ms, mean baseline EPSP slope before pairings was 0.95 ± 0.55 mV/ms and 0.98 ± 0.46 mV/ms one hour after pairings; variation 3%).

Taken together, there was no significant change in synaptic weight after post-pre pairings (95 ± 5%, *p* = 0.3611, n = 23), but tLTD was observed with pre-post pairings (88 ± 4%, *p* = 0.0278, n = 12). However, if we considered separately tLTP and tLTD, post-pre pairings induced mainly tLTD (mean value of the EPSP slope recorded 60 min after STDP protocol: 71 ± 5%, n = 9, *p* = 0.0089) and pre-post pairings induced exclusively tLTD (77 ± 3%, n = 6, *p* = 0.0340) (Fig. 2g). The temporal window of tLTD induction was large, spanning over 200 ms (−100 < Δt_STDP_ < +100 ms). Beyond Δt_STDP_ ±100 ms, no significant plasticity was induced (101 ± 1%, n = 3, *p* = 0.7500).

In addition to tLTD dominance, it should be noted that almost half of the STDP pairings did not induce significant plasticity (failure rate: 43%, n = 35). Indeed, 39% (n = 23) and 50% (n = 12) of the post-pre and pre-post pairing experiments, respectively, failed to induce long-term synaptic efficacy changes (post-pre pairings: 99 ± 3%, n = 9, *p* = 0.8583 and pre-post pairings: 99 ± 3%, n = 6, *p* = 1.000).

In some cases (22%, 5/23 post-pre pairing experiments), we observed tLTP for post-pre pairings in a narrow Δt_STDP_ range (−30 < Δt_STDP_ < 0 ms) (Fig. 2d). Among the 14 experiments performed within −30 < Δt_STDP_ < 0 ms, we observed a mixture of plasticity with similar occurrence (36%) between tLTD (67 ± 8%, n = 5, *p* = 0.0179) and tLTP (132 ± 6%, n = 5, *p* = 0.0425), as well as no plasticity (28%) (105 ± 3%, n = 4, *p* = 0.2500) (Fig. 2g). Interestingly, we found a significant correlation (r^2^ = 0.41) between the number of postsynaptic action potentials evoked during the post-pre pairings and the polarity of plasticity (for −30 < Δt_STDP_ < 0 ms). Indeed, more than one post-synaptic spike favoured induction of tLTP (2.4 ± 0.2 spikes, n = 3), whereas one spike was associated to tLTD expression (1.2 ± 0.2 spikes, n = 5) (*p* = 0.0323) (Fig. 2i). This correlation between the number of post-synaptic spikes during the STDP pairings and the polarity of plasticity was found exclusively for narrow negative Δt_STDP_ (*i.e*. −30 < Δt_STDP_ < 0 ms), since for more negative Δt_STDP_ (−100 < Δt_STDP_ < −30 ms) and positive Δt_STDP_ (0 < Δt_STDP_ < +100 ms), only tLTD or absence of plasticity was observed, independent of the number of post-synaptic spikes (range from 1 to 3 spikes; r^2^ = 0.65).

It was reported in *ex vivo* studies that induction of STDP depends on EPSP magnitude (resulting from pre-synaptic stimulation)^36^. Here, there was no correlation between tLTD and tLTP induction after post-pre pairings with the EPSP amplitudes (r^2^ = 0.096), Vm (r^2^ = 0.0001), Ri (r^2^ = 0.0058) or rheobase (r^2^ = 0.2456; tLTP: 0.54 ± 0.04 nA, n = 5, and tLTD: 0.43 ± 0.05 nA, n = 9; *p* = 0.1758) of the recorded MSNs (n = 14). We investigated whether Vm during baseline, STDP pairings or during the last five minutes of recordings influences the polarity of plasticity and magnitude (Supplementary Fig. 1). In SE and deep anaesthesia conditions, we did not find any correlation between Vm (mean or variance) and plasticity output, except for the magnitude of tLTD induced by pre-post pairings. Plots of Vm during the STDP pairings are illustrated for each representative experiment (Fig. 2c–f). In conclusion, Vm fluctuations (including during the STDP pairings) do not account for the plasticity polarity (Supplementary Fig. 1).

Thus, in *in vivo* deeply anaesthetised adult rats grown in SE, we mainly observed tLTD induced with low success rate in a wide Δt_STDP_ range (~200 ms), in contrast to what has been described in *ex vivo* corticostriatal STDP experiments showing that bidirectional STDP was induced in a much shorter temporal window (~60 ms: −30 < Δt_STDP_ < +30 ms) with lower failure rate (<15%)^16,17,34,37–40^.

### Anaesthesia with chloral hydrate favours tLTD

The previous experiments were performed in anaesthetised adult animals. In contrast, *ex vivo* studies, which widely reported bidirectional STDP centred on Δt_STDP_ = 0 in various brain structures^11^, were generally performed in juvenile animals without anaesthetics. Thus, we tested whether age and anaesthesia status may influence STDP. For this purpose, we performed experiments in brain slices from P_65-90_ rats, because it is not possible to perform *in vivo* long-duration (*i.e*. >80 min) intracellular recordings in non-anaesthetised animals.

Using whole-cell patch-clamp recordings in horizontal brain slices (Fig. 3a,b), we found bidirectional STDP in a restricted time window (~60 ms). As previously reported for juvenile rodents at corticostriatal synapses^34,38,39,41^, we induced bidirectional STDP centred on Δt_STDP_ = 0 ms with 100 pairings (n = 40). Figure 3c,d show examples of tLTP and tLTD induced by 100 post-pre and pre-post pairings, respectively. Post-pre pairings (−30 < Δt_STDP_ < 0 ms) induced tLTP (146 ± 15%, *p* = 0.0004, n = 9; success rate: 69%), whereas pre-post pairings (0 < Δt_STDP_ < +30 ms) induced tLTD (67 ± 5%, *p* = 0.0039, n = 9; success rate: 90%) (Fig. 3e). Pairings for −60 < Δt_STDP_ < −35 ms and +30 < Δt_STDP_ < +40 ms did not induce plasticity (105 ± 5, *p* = 0.0823, n = 5, and 101 ± 6, *p* = 0.6589, n = 7, respectively) (Fig. 3e). Thus, polarity and magnitude of STDP were similar between juvenile and adult animals. We recorded STDP in the absence of GABA_A_ receptor antagonist, because we previously showed that GABA operates as a Hebbian/anti-Hebbian switch at corticostriatal synapses^16,17^; thus anti-Hebbian polarity is preserved, as observed *in vivo* at corticostriatal synapses^42^. Anaesthesia affects neuronal properties and excitation/inhibition balance^43,44^. We then tested whether anaesthesia would affect STDP features. For this purpose, we studied the effect of trichloroethanol, the major metabolite of chloral hydrate responsible for anaesthesia^45^ and the anaesthetic used to perform the *in vivo* recordings in this study. We first investigated the effects of bath-applied trichloroethanol (5 mM) on corticostriatal transmission and found a marked decrease in the amplitude of EPSC (by 47.4 ± 6.2%, *p* < 0.0001, n = 17). We then investigated the effects of trichloroethanol on STDP and observed a unidirectional STDP in which only tLTD was induced. As exemplified in Fig. 3f,g, post-pre and pre-post pairings induced tLTD. To summarize, 100 post-pre pairings induced tLTD (65 ± 5%, *p* = 0.0020, n = 10; success rate: 100%) as well as pre-post pairings (48 ± 7%, *p* = 0.0425, n = 5; success rate: 100%) (Fig. 3h). In addition, Δt_STDP_ was considerably larger under trichloroethanol for both post-pre and pre-post pairings (−100 < Δt_STDP_ < +100 ms) (Fig. 3h) in comparison with control conditions (−30 < Δt_STDP_ < +30 ms) (Fig. 3e). Therefore, in corticostriatal brain slices, trichloroethanol treatment prevents tLTP induction to the benefit of tLTD with increased Δt_STDP_.

**Figure 4:**
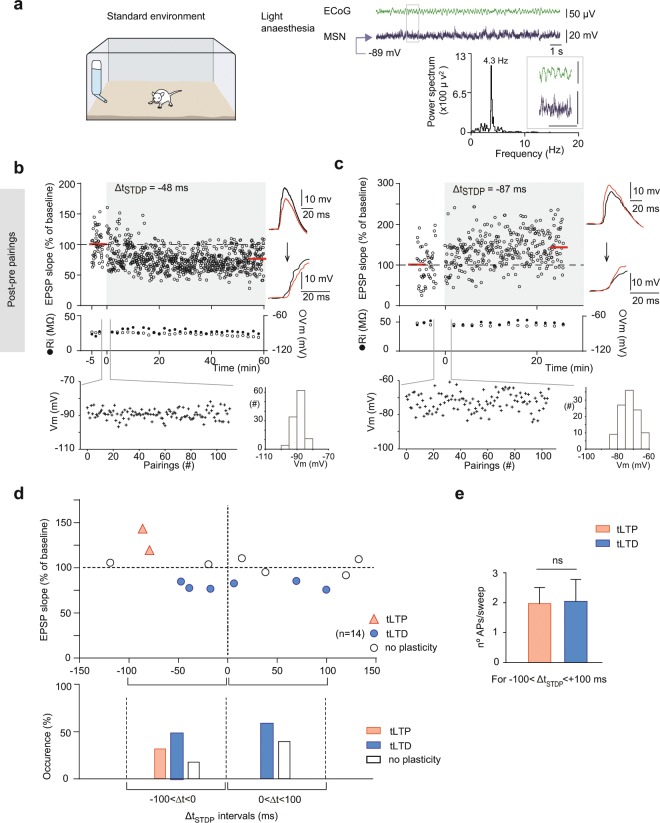
Light anaesthesia status promotes *in vivo* corticostriatal bidirectional STDP. (**a**) Top-right, intracellular recording of the spontaneous activity of a single MSN (Vm = −89 mV) together with the corresponding ECoG waves in lightly anaesthetised rats under painless contention (note the attenuation of up and down states in comparison with that shown in deeply anaesthetised rats (Fig. 2a); bottom-right, spectral analysis of the ECoG recordings showing a strong periodicity at 4.3 Hz associated with faster waves, corresponding to a light level of anaesthesia. (**b**,**c**) Representative experiments illustrating the time courses of synaptic efficacy changes induced by STDP protocols. Bottom panels: evolution of input resistance (Ri) (●) and Vm (○) illustrates the stability of the recordings along time (averaged every 2 min). Representative traces are the average of 60 EPSPs during baseline (black traces) and 55–60 min after STDP pairings (red traces). (**b**) Example of tLTD induced by 100 post-pre pairings with Δt_STDP_ = −48 ms. Top, EPSP slope before and after 100 pairings (before pairings: 2.66 ± 0.09 mV/ms; 55–60 min after pairings: 2.05 ± 0.10 mV/ms; decrease of 77%). Bottom, time courses of Ri (before, 24.0 ± 1.7 MΩ; after, 25. 7 ± 1.2 MΩ; change of 7%) and Vm (before, −89.0 ± 0.6 mV; after, −96. 7 ± 0.3 mV; change of 9%) for this cell; Vm is also shown during STDP pairings (Vm just before each pairing as a function of the number of pairings). (**c**) Example of tLTP induced by 100 post-pre pairings with Δt_STDP_ = −87 ms. Top, EPSP slope before and after 100 pairings (before pairings: 0.80 ± 0.05 mV/ms; 55–60 min after pairings: 1.21 ± 0.08 mV/ms; increase of 153%). Bottom, time courses of Ri (before, 48.3 ± 2.0 MΩ; after, 48.0 ± 0.6 MΩ; change of 1%) and Vm (before, −72.7 ± 1.2 mV; after, −76.0 ± 0.6 mV; change of 5%) for this cell; Vm is also shown during STDP pairings (Vm just before each pairings as a function of the number of pairings). (**d**) Summary of STDP experiments (n = 14) showing a dominance of tLTD with a high rate of failure of plasticity induction and tLTP expression in a restricted Δt_STDP_ without overlapping with Δt_STDP_ of tLTD (−30 < Δt_STDP_ < 0 ms). (**e**) No significant correlation between the number of post-synaptic action potentials evoked during STDP pairings and the polarity of plasticity (*p* = 0.3626, unpaired Student *t*-test).

### Light anaesthesia status favours *in vivo* bidirectional plasticity

The use of long-duration intracellular recordings (sharp pipette electrode) prevents any investigation on freely-moving animals because of mechanical instability. In order to reduce, as much as possible, the level of anaesthesia, we performed long-duration intracellular recordings in lightly anaesthetised rats in painless contention; in this series of experiments rats were grown in SE. The light anaesthesia state was controlled by recording spontaneous ECoG activity and was adjusted until the ECoG spectral analysis showed a dominant frequency ~ 3–5 Hz range associated with faster waves (Fig. 4a). In parallel, the up and down states of the MSN Vm observed in deeply anaesthetised rats disappeared under light anaesthesia (down to up fluctuations of 16 ± 1 mV) (Table 1). In these conditions, we obtained long-duration recordings in 14 MSNs.

In rats reared in SE, we observed a different STDP map in light anaesthesia compared to deep anaesthesia. Indeed, although tLTD with a high rate of failure of plasticity induction was still dominant, we found that few tLTP could be induced for a restricted Δt_STDP_ without overlapping with tLTD domain of expression (for −100 < Δt_STDP_ < +100 ms: success rate of tLTD: 55%, tLTP: 18% and no plasticity: 27%; n = 11) (Fig. 4d). Moreover, post-pre pairings with −50 < Δt_STDP_ < 0 ms (n = 4) induced tLTD (76 ± 0.3%; success rate: 75%), whereas post-pre pairings with −100 < Δt_STDP_ < −50 ms (n = 3) induced tLTP (131 ± 12%, n = 2; success rate: 67%) (Fig. 4d). Thus, with post-pre pairings a bidirectional STDP was observed, with tLTD and tLTP occurring in distinct Δt_STDP_. As exemplified in Fig. 4b,c, post-pre pairings (Δt_STDP_ = −48 ms) induced tLTD (mean baseline EPSP slope, 2.66 ± 0.09 mV/ms, decreased by 77%, to 2.05 ± 0.10 mV/ms, one hour after pairing), whereas tLTP was induced for larger Δt_STDP_ (for Δt_STDP_ = −87 ms, mean baseline EPSP slope, 0.80 ± 0.05 mV/ms, increased by 153%, to 1.21 ± 0.08 mV/ms, 26 min after pairing). Pre-post pairings for 0 < Δt_STDP_ < +100 ms (n = 5) triggered tLTD (79 ± 4%, n = 3; success rate: 60%) (Fig. 4d). Beyond Δt_STDP_ = ±100 ms, no significant plasticity was observed (102 ± 5%, *p* = 1.0000, n = 3).

Thus, under light anaesthesia bidirectional STDP was observed on the post-pre pairing side, while tLTD still dominated in large Δt_STDP_. We did not find significant difference between the polarity of plasticity and the number of post-synaptic spikes (*p* = 0.3626) (Fig. 4e). We did not find significant correlation between Vm (mean or variance) and plasticity output, (except for the expression of tLTP induced by post-pre pairings, which could be biased due to the low numbers of tLTP) (Supplementary Fig. 2). Plots of Vm during the STDP pairings are illustrated for representative experiments (Fig. 4b,c).

STDP recorded in deep *versus* light anaesthetised rats grown in SE display common features with overall tLTD dominance occurring in wide Δt_STDP_ (~200 ms). However, a bidirectional trend of STDP (*i.e*. tLTP and tLTD occurring in distinct Δt_STDP_) emerges in light anaesthesia, whereas in deep anaesthesia tLTP could be also observed, but sharing the same Δt_STDP_ as tLTD. We found a similar failure rate of plasticity induction between deep and light anaesthesia conditions (42 *vs* 43%) for −150 < Δt_STDP_ < +150 ms. When restricted to −100 < Δt_STDP_ < +100 ms, a higher failure rate was observed in deep than in light anaesthesia conditions (37% *vs* 27%).

### Environmental enrichment promotes bidirectional *in vivo* plasticity (centred on Δt_STDP_ = −50 ms)

**Figure 5:**
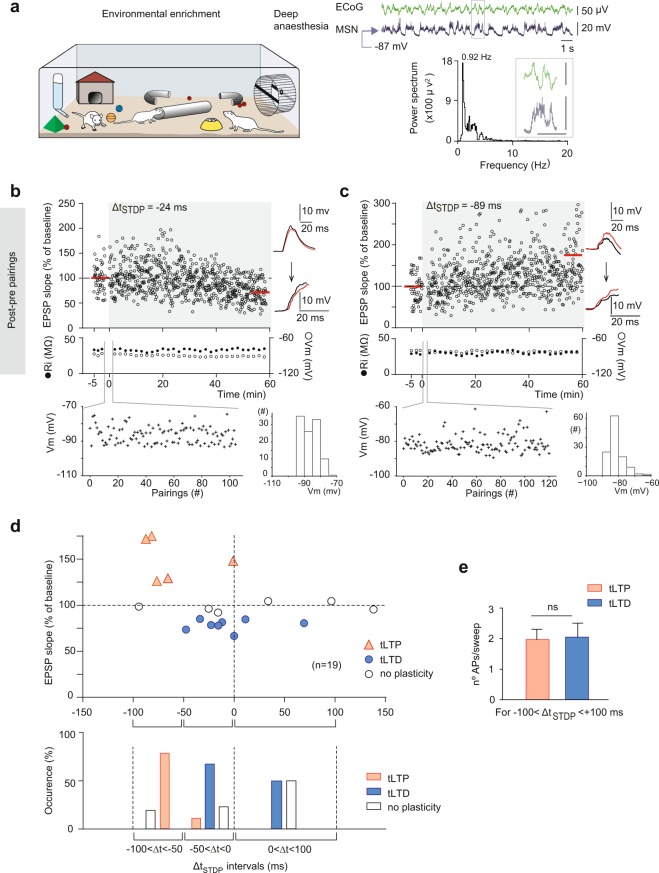
Environmental enrichment favours *in vivo* corticostriatal bidirectional STDP for post-pre pairings centred on Δt_STDP_ = −50 ms. (**a**) Top-right, intracellular recording of the spontaneous activity of a single MSN (Vm = −87 mV) together with the corresponding ECoG waves showing the characteristic up and down states observed in deeply anaesthetised rats; bottom-right, spectral analysis of the ECoG recordings showing a strong periodicity at 0.92 Hz corresponding to a deep level of anaesthesia. (**b**,**c**) Representative experiments illustrating the time courses of synaptic efficacy changes induced by STDP protocols. Bottom panels: evolution of input resistance (Ri) (●) and Vm (○) illustrates the stability of the recordings along time (averaged every 2 min). Representative traces are the average of 60 EPSPs during baseline (black traces) and 55–60 min after STDP pairings (red traces). (**b**) Example of tLTD induced by 100 post-pre pairings with Δt_STDP_ = −24 ms. Top, EPSP slope before and after 100 pairings (before pairings: 1.33 ± 0.05 mV/ms; 55–60 min after pairings: 1.04 ± 0.07 mV/ms; decrease of 78%). Bottom, time courses of Ri (before, 33.0 ± 0.6 MΩ; after, 34.3 ± 0.7 MΩ; change of 4%) and Vm (before, −87.7 ± 1.2 mV; after, −91.0 ± 0.6 mV; change of 4%) for this cell; Vm is also shown during STDP pairings (Vm just before each pairings as a function of the number of pairings). (**c**) Example of tLTP induced by 100 post-pre pairings with Δt_STDP_ = −89 ms. Top, EPSP slope before and after 100 pairings (before pairings: 0.62 ± 0.053 mV/ms; 26 min after pairings: 1.07 ± 0.06 mV/ms; increase of 172%). Bottom, time courses of Ri (before, 29.0 ± 0.6 MΩ; after, 31.7 ± 0.3 MΩ; change of 9%) and Vm (before, −79.7 ± 0.3 mV; after, −82.7 ± 0.3 mV; change of 4%) for this cell; Vm is also shown during STDP pairings (Vm just before each set of pairings as a function of the number of pairings). (**d**) Summary of STDP experiments (n = 19) in EE rats showing strict orientation with distinct Δt_STDP_ for tLTD (−50 < Δt_STDP_ < 0 ms) or tLTP (−100 < Δt_STDP_ < −50 ms) induction and centred on Δt_STDP_ = −50 ms. (**e**) No significant correlation between the number of post-synaptic action potentials evoked during STDP pairings and the polarity of plasticity (*p* = 0.4963, unpaired Student *t*-test).

Acquisition of a task involving procedural learning has been associated with an increased AMPA/NMDA ratio, i.e. LTP, at corticostriatal synapses^25,27–30,46^. Due to increased learning and memory performance reported in rodents raised in EE^1–5^, we tested whether rearing in EE could promote tLTP, as opposed to the tLTD dominance (and the high failure rate of plasticity) observed in rats grown in SE. To this purpose, 19 MSNs were recorded *in vivo* in EE rats under deep anaesthesia. The level of anaesthesia was adjusted until the ECoG spectral analysis showed a high-power dominant frequency in the ~1–3 Hz range (Fig. 5a).

Rats grown in EE and SE exhibited different STDP maps. First, STDP post-pre pairings induced long-term synaptic efficacy changes with a success rate of 79% (n = 14), denoting less failure in plasticity induction than in SE rats recorded under deep or light anaesthesia (success rate: 61%, n = 23 *vs* 71%, n = 7, respectively). *In vivo* intracellular recordings (n = 19) performed in rats grown in EE revealed a bidirectional STDP centred on Δt_STDP_ = −50 ms (Fig. 5). Indeed, post-pre pairings with −50 < Δt_STDP_ < 0 ms (n = 9) induced mainly tLTD (79 ± 3%, n = 6, *p* = 0.0355) or an absence of plasticity (93 ± 2%, n = 2), whereas for a wider temporal window (−100 < Δt_STDP_ < −50 ms) tLTP was exclusively observed (151 ± 13%, n = 4, *p* = 0.0467) (Fig. 5d). As shown in Fig. 5b,c, post-pre pairings (Δt_STDP_ = −24 ms) induced tLTD (mean baseline EPSP slope, 1.33 ± 0.05 mV/ms, decreased by 78%, to 1.04 ± 0.07 mV/ms, one hour after pairing), as well as tLTP (Δt_STDP_ = −89 ms) (mean baseline EPSP slope, 0.62 ± 0.03 mV/ms, increased by 172%, to 1.07 ± 0.06 mV/ms, one hour after pairing). In this set of experiments, the occurrence of tLTP or tLTD was not dependent on the number of post-synaptic spikes emitted during the STDP protocol (*p* = 0.4963) (Fig. 5e). While bidirectional STDP was observed for post-pre pairings, plasticity was hardly elicited (94 ± 5%, n = 5, *p* = 0.2785) for pre-post pairings (0 < Δt_STDP_ < +150 ms); among these 5 MSNs, two tLTD were induced (Fig. 5d). We did not find a correlation between Vm (mean or variance) and plasticity output (Supplementary Fig. 3). Plots of Vm during the STDP pairings are illustrated for representative experiments (Fig. 5b,c).

In EE rats, STDP exhibited a bidirectional STDP centred on Δt_STDP_ = −50 ms, i.e. strictly oriented on either side of −50 ms with distinct Δt_STDP_ for tLTP (−100 < Δt_STDP_ < −50 ms) and tLTD (−50 < Δt_STDP_ < 0 ms). As pre-post pairings mainly failed inducing plasticity, the temporal window of plasticity induction was restricted to post-pre pairings and spanned over 100 ms, i.e. half the width of STDP observed in SE rats. These results indicate that EE promotes bidirectional STDP, in distinct Δt_STDP_, centred around −50 ms in a shorter Δt_STDP_ and with a higher success rate than STDP observed in SE rats.

### Environmental enrichment associated with light anaesthesia promotes bidirectional STDP (centred on Δt_STDP_ = 0 ms)

**Figure 6:**
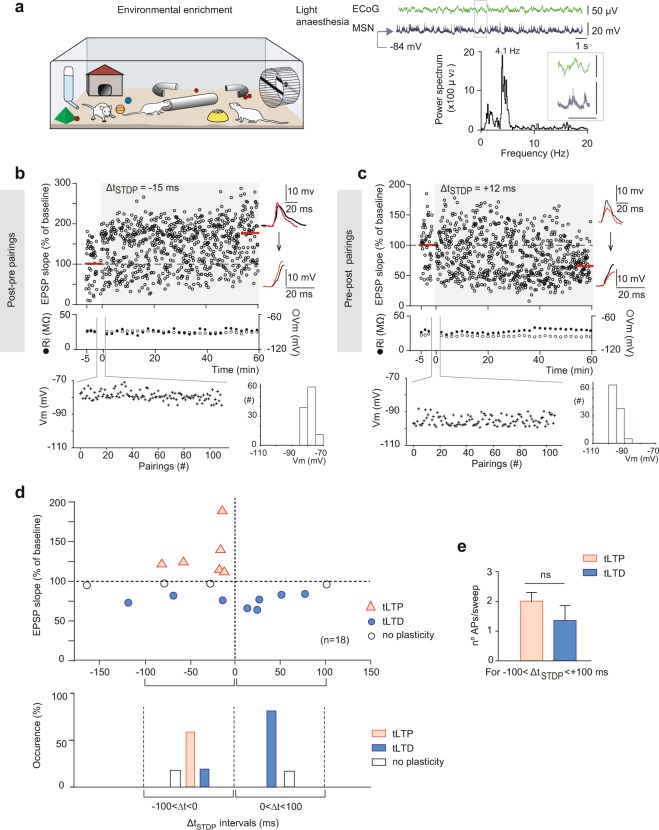
Rats reared in EE and recorded under light anaesthesia show *in vivo* corticostriatal bidirectional STDP centred on Δt_STDP_ = 0 ms. (**a**) Top-right, intracellular recording of the spontaneous activity of a single MSN (Vm = −84 mV) together with the corresponding ECoG waves in EE rats recorded under light anaesthesia (note the attenuation of up and down states in comparison with that shown in deeply anaesthetised rats (Fig. 2a); bottom-right, spectral analysis of the ECoG recordings showing a strong periodicity at 4.1 Hz corresponding to a light level of anaesthesia. (**b**,**c**) Representative experiments illustrating the time courses of synaptic efficacy changes induced by STDP protocols. Bottom panels: evolution of input resistance (Ri) (●) and Vm (○) illustrates the stability of the recordings along time (averaged every 2 min). Representative traces are the average of 60 EPSPs during baseline (black traces) and 55–60 min after STDP pairings (red traces). (**b**) Example of tLTP induced by 100 post-pre pairings with Δt_STDP_ = −15 ms. Top, EPSP slope before and after 100 pairings (before pairings: 1.44 ± 0.10 mV/ms; 55–60 min after pairings: 2.52 ± 0.20 mV/ms; increase of 175%). Bottom, time courses of Ri (before, 25.3 ± 0.3 MΩ; after, 28.0 ± 0.6 MΩ; change of 11%) and Vm (before, −88.0 ± 0.6 mV; after, −90.7 ± 0.3 mV; change of 3%) for this cell; Vm is also shown during STDP pairings (Vm just before each pairings as a function of the number of pairings). (**c**) Example of tLTD induced by 100 pre-post pairings with Δt_STDP_ = +12 ms. Top, EPSP slope before and after 100 pairings (before pairings: 2.57 ± 0.10 mV/ms; 55–60 min after pairings: 1.70 ± 0.07 mV/ms; decrease of 66%). Bottom, time courses of Ri (before, 28.3 ± 0.9 MΩ; after, 29.7 ± 0.3 MΩ; change of 5%) and Vm (before, −92.3 ± 0.7 mV; after, −95.7 ± 0.3 mV; change of 4%) for this cell Vm is also shown during STDP pairings (Vm just before each set of pairings as a function of the number of pairings). (**d**) Summary of STDP experiments (n = 18) showing bidirectional STDP centred on Δt_STDP_ = 0 ms. Post-pre pairings (−100 < Δt_STDP_ < 0 ms) induced mainly tLTP, whereas pre-post pairings (0 < Δt_STDP_ < 100 ms) triggered exclusively tLTD. (**e**) No significant correlation between the number of post-synaptic action potentials evoked during STDP pairings and the polarity of plasticity (*p* = 0.3206, unpaired Student *t*-test).

We next tested the combined effects of EE and light anaesthesia by recording long-term synaptic efficacy changes in 18 MSNs. As mentioned earlier, the proper level of anaesthesia was assessed according to recordings of ECoG activity and was adjusted until the ECoG spectral analysis showed a dominant frequency ~3–5 Hz range (Fig. 6a).

In these conditions, we found a bidirectional STDP centred on 0 ms, suggesting that tLTP and tLTD were induced by distinct pairing polarity: tLTP and tLTD were triggered by post-pre and pre-post pairings, respectively. Indeed, post-pre pairings (−100 < Δt_STDP_ < 0 ms) induced mainly tLTP (140 ± 14%, *p* = 0.0313, n = 6), whereas pre-post pairings (0 < Δt_STDP_ < 100 ms) triggered exclusively tLTD (75 ± 4%, *p* = 0.0425, n = 5) (Fig. 6). A few tLTDs were also observed for post-pre pairings (−100 < Δt_STDP_ < 0 ms, 74 ± 2%, n = 2). Figure 6b shows an example of tLTP induced by 100 post-pre pairings (Δt_STDP_ = −15 ms); the mean baseline EPSP slope was 1.44 ± 0.10 mV/ms and increased by 175% to 2.52 ± 0.20 mV/ms one hour after pairings. Pre-post pairings induced only tLTD as exemplified in Fig. 6c; 100 pre-post pairings (Δt_STDP_ = +12 ms) induced tLTD, where the mean baseline EPSP slope was 2.57 ± 0.10 mV/ms before pairings and decreased by 66% to 1.70 ± 0.07 mV/ms one hour after pairings.

Here, the occurrence of tLTP or tLTD was not dependent on the number of post-synaptic spikes emitted during the STDP protocol (*p* = 0.3206) (Fig. 6e). We did not find a correlation between Vm (mean or variance) and plasticity output (Supplementary Fig. 4). Plots of Vm during STDP pairings are illustrated for representative experiments (Fig. 6b,c).

Beyond Δt_STDP_ = ± 100 ms no significant plasticity was observed (88 ± 8%, n = 3, *p* = 0.3248). The success rate of plasticity induction was increased in EE rats recorded under light anaesthesia; 87% (n = 15) for −100 < Δt_STDP_ < +100 ms compared to other conditions described earlier (SE rats under deep or light anaesthesia or EE rats under deep anaesthesia). Moreover, the failure rate of plasticity induction dropped to null for tLTD induced by pre-post pairings (0 < Δt_STDP_ < 100 ms, n = 5). For post-pre pairings, the results were more complex. Indeed, the failure rate of plasticity induction was considerably reduced in EE rats under light anaesthesia (25%, n = 10) with 50% of tLTP, 25% of tLTD and 25% absence of plasticity (Fig. 6d). Therefore, EE conditions coupled to light anaesthesia allow occurrence of an anti-Hebbian bidirectional STDP, as reported *in vitro* without blockade of GABA_A_ transmission^16,17^.

### Comparison of STDP in rats reared in SE or EE, and recorded in deep or light anaesthesia

**Figure 7:**
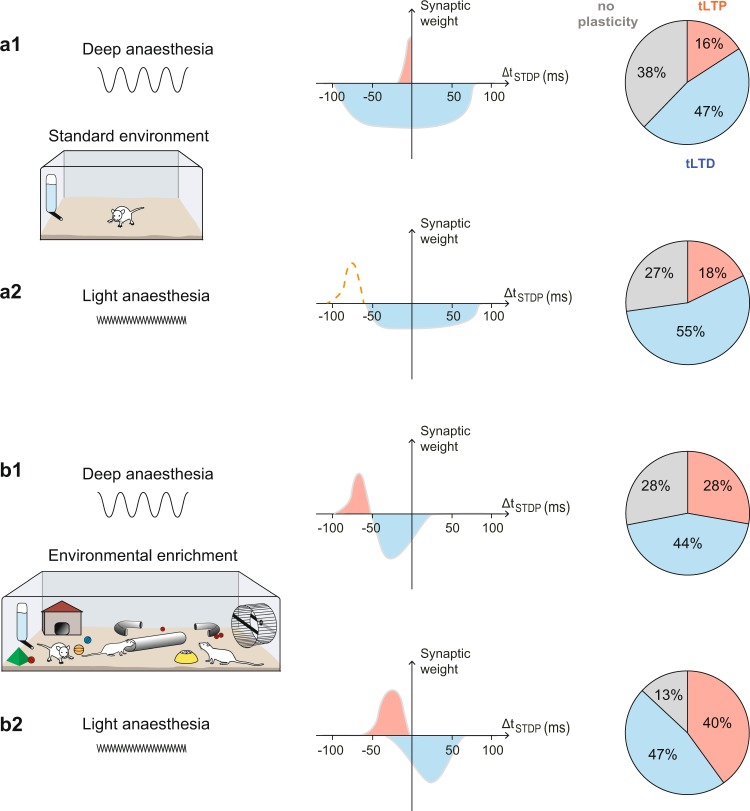
Schematic representation of the control exerted by rearing environment and anaesthetic regime on striatal STDP *in vivo*. Adult rats reared in SE (**a1**,**a2**) exhibit a dominance of tLTD over tLTP with a plasticity expression within a large Δt_STDP_ (~200 ms), whereas animals grown in EE (**b1**,**b2**) exhibit bidirectional anti-Hebbian STDP confined in more restricted Δt_STDP_ (~100 ms). Deep anaesthesia (**a1**,**b1**) triggers a higher rate of failure for plasticity induction. EE housing favours a higher rate of tLTP expression together with a lower occurrence of absence of plasticity (upon STDP pairings). Across conditions, tLTD occurrence appears stable. Combined effects of EE and light anaesthesia (**b2**) promote anti-Hebbian cortico-striatal STDP with an asymmetrical feature such as post-pre pairings and pre-post induced mainly tLTP and tLTD, respectively, in a restricted temporal window (~100 ms). Pie charts indicates the proportion of tLTP, tLTD and failure occurrences. The dashed line in panel (a2) indicates only a tendency because of the low number of STDP collected in this condition (SE in light anaesthesia).

Differences, in terms of plasticity, were observed depending on the four conditions considered here: rats grown in SE or EE and recorded under deep or light anaesthesia (Fig. 7). EE promotes induction of bidirectional asymmetric plasticity, which was not observed in SE rats deeply anaesthetised. Although the light anaesthetic regime (even in rats reared in SE) tends also to allow for the emergence of bidirectional plasticity, this plasticity was not centred on 0 ms since the tLTP/tLTD transition occurred around −50 ms. In contrast to SE conditions, in which tLTD dominates and tLTP occurrence is lower than the failure of plasticity (SE & deep anaesthesia: 47% tLTD, 16% tLTP, 38% no plasticity, n = 32; SE & light anaesthesia: 55% tLTD, 18% tLTP, 27% no plasticity, n = 11), in EE rats (regardless of the anaesthetic regime) tLTP occurrence is larger than the failure rate of plasticity, while tLTD remains stable (EE & deep anaesthesia: 44% tLTD, 28% tLTP, 28% no plasticity, n = 18; EE & light anaesthesia: 47% tLTD, 40% tLTP, 13% no plasticity, n = 15); here, we considered data harvested for −100 < Δt_STDP_ < +100 ms.

We did not find a significant correlation between basal properties and expression and polarity of plasticity (r^2^ = 0.04, *p* = 0.1419), input resistance (r^2^ = 0.02, *p* = 0.3235), rheobase (r^2^ = 3 × 10^−6^, *p* = 0.9712), EPSP amplitude (r^2^ = 0.07, *p* = 0.5342) (Table 1). For MSNs recorded within 100 < Δt_STDP_ < +100 ms (SE & deep anaesthesia, n = 32; SE & light anaesthesia, n = 11; EE & deep anaesthesia, n = 18; EE & light anaesthesia, n = 16), there was no significant difference in input resistance (Kruskal-Wallis test, *p* = 0.0661), rheobase (Kruskal-Wallis test, *p* = 0.9901) or EPSP amplitude (Kruskal-Wallis test, *p* = 0.8747), except for Vm (one-way ANOVA, *p* = 0.0463, SE & deep anaesthesia vs EE & light anaesthesia). As mentioned earlier, there was no correlation between the number of action potentials and plasticity, except for post-pre pairings STDP in SE rats under deep anaesthesia; in this latter case, tLTP was favoured by a higher number (i.e. 2 *versus* 1) of back-propagating action potentials (Fig. 2i); the number of post-synaptic action potentials was not significantly different across the four experimental conditions (SE & deep anaesthesia 2.0 ± 0.2 (n = 11), SE & light anaesthesia 2.4 ± 0.4 (n = 5), EE & deep anaesthesia 2.2 ± 0.5 (n = 8), EE & light anaesthesia 1.9 ± 0.2 (n = 8), one-way ANOVA, *p* = 0.7398). Moreover, the evoked amplitude of tLTP and tLTD was not different across conditions (tLTP: SE & deep anaesthesia 132 ± 6%, SE & light anaesthesia 136 ± 17%, EE & deep anaesthesia 150 ± 10%, EE & light anaesthesia 140 ± 14%, Kruskal-Wallis test, *p* = 0.5776; tLTD: SE & deep anaesthesia 73 ± 3%, SE & light anaesthesia 78 ± 2%, EE & deep anaesthesia 79 ± 2%, EE light anaesthesia 75 ± 3%, Kruskal-Wallis test, *p* = 0.4976).

Failure rate was high (38%) in SE rats in deep anaesthesia. Interestingly, the occurrence of failure in induced plasticity (tLTP or tLTD) within –100 < Δt_STDP_ < 100 ms was the lowest in EE rats under light anaesthesia (failure rate: 13%), whereas it was similar between rats grown in SE under light anaesthesia and EE under deep anaesthesia (27 and 28%, respectively) (Fig. 7).

EE or light anaesthesia tended to reduce the failure rate accompanied with the emergence of bidirectional STDP (not centred on 0 ms), whereas EE and light anaesthetic regime diminished the failure rate and favoured bidirectional STDP centred on 0 ms. Light anaesthetic regime (when compared to deep anaesthesia) decreased the failure rate of plasticity, whereas EE (when compared to SE) favoured tLTP and decreased the failure rate of plasticity; tLTD occurrence appeared stable across the four conditions examined here (Fig. 7).

## Discussion

In this study, we show that *in vivo* polarity and characteristics of STDP in the dorsolateral striatum vary as a function of housing conditions (SE *vs* EE) and the anaesthetic regime. Indeed, for rats housed in SE and recorded under deep anaesthesia, tLTD constitutes the main form of plasticity regardless of the order of pre- and post-synaptic activity. This is in agreement with previous *in vivo* studies reporting tLTD as the main form of STDP or absence of plasticity at corticostriatal synapses in anaesthetised (urethane) rodents grown in SE^42,47^. Here, occasionally some tLTP were observed for post-pre pairings (−30 < Δt_STDP_ < 0 ms) and were favoured by a higher number of post-synaptic action potentials. It remains to be examined whether these tLTPs are preferentially expressed in striatopallidal MSNs (D_2_R-expressing MSNs) than in striatonigral MSNs (D_1_R-expressing MSNs). D_2_R-MSNs are more excitable than D_1_R-MSNs^48^, and they show higher dendritic excitability than D_1_R-MSN, allowing back-propagating action potentials to invade more efficiently in distal dendrites in D_2_R-MSNs^49^. We found that deep anaesthesia (chloral hydrate) prevented tLTP expression to the benefit of tLTD (i.e. symmetric anti-Hebbian tLTD was observed). Therefore, shifting from deep to light anaesthetic regime in rats grown in SE seems to favour bidirectional STDP for Δt_STDP_ < 0 ms, but still with a majority of tLTD induction and failure of plasticity; due to the relatively low number of experiments in SE rats under light anaesthesia, further research is required to firmly determine the occurrence of tLTP for post-pre pairings (−100 < Δt_STDP_ < −50 ms). These findings were clearer when rats were grown in EE, even under deep anaesthesia: we observed STDP quasi-exclusively for post-pre pairings with tLTP and tLTD being expressed in distinct Δt_STDP_ domains, *i.e*. −100 < Δt_STDP_ < −50 ms and −50 < Δt_STDP_ < 0 ms, respectively. Importantly, when *in vivo* intracellular recordings were performed in EE rats under light anaesthesia, bidirectional and asymmetric anti-Hebbian STDP was observed: tLTP and tLTD were induced by post-pre and pre-post pairings, respectively. *In vivo* corticostriatal STDP could be induced in larger Δt_STDP_ than in brain slices (~150 ms *in vivo*: −50 < Δt_STDP_ < +100 ms *vs* ~70 ms *ex vivo*: −30 < Δt_STDP_ < +40 ms)^34,37–40^. Interestingly, in SE conditions, regardless of the anaesthetic regime, the occurrence of tLTP is similar (16 and 18%, in deep and light anaesthesia, respectively), whereas in EE the anaesthetic regime displays a greater influence on tLTP expression (28% and 40%, in deep and light anaesthesia, respectively) (Fig. 7). Therefore, this indicates that housing conditions exert a marked influence (larger than the anaesthetic regime) on map plasticity.

Since variability is common in synaptic plasticity, effects of EE on STDP may be variable. Indeed, the effect of EE on STDP is expected to be different depending on the brain area (cortex, hippocampus, cerebellum, striatum), on the various forms of STDP (Hebbian or anti-Hebbian, symmetric or asymmetric, bidirectional or unidirectional) and also on the signalling pathways (for example, NMDA receptors (NMDAR) and/or endocannabinoids)^11^. Interestingly, NMDAR- and endocannabinoid-mediated STDP are differentially impacted by noisy spike timings (mimicked by jittered pairings): NMDAR-tLTP being susceptible and endocannabinoid-tLTD or -tLTP being robust to jitter^50^. Here, using STDP pairings with fixed Δt_STDP_, we did not observe jitter >3 ms. Therefore, it is less likely that (NMDAR)-tLTP could not be observed because of spike jittering. However, under *in vivo* physiological patterns, we can hypothesize that tLTP would be more difficult to be induced because of large jitters, whereas endocannabinoid-STDP would emerge more easily. The underlying signalling pathways of *in vivo* striatal tLTP and tLTD remain also to be characterised. Interestingly, an increase in the AMPA/NMDA ratio, a proxy for LTP, has been detected in awake and behaving mice over-trained to accomplish a task involving procedural learning^25–30,46^. *In vivo* investigation of synaptic plasticity mostly performed in adult and anaesthetised rodents, showed that LTD was easily elicited while LTP was hardly observed. As an illustration, in *in vivo* anaesthetised mice, corticostriatal tLTP was induced only when combined with electrical stimulation of the dopaminergic neurons of the substantia nigra *pars compacta* or with pharmacological blockade of GABAergic transmission in the midbrain superior colliculus^47^.

In the neocortex or the hippocampus, several *ex vivo* studies from animals subjected to EE have reported changes in the excitation/inhibition balance, serotoninergic or cholinergic tones, increase of noradrenaline or BDNF, enhanced LTP and LTD, increase in mEPSCs amplitude and frequency, changes in neuronal excitability or increase in gamma oscillations. It is fair to note that an equivalent amount of studies have shown opposite results or the absence of significant changes for some of the aforementioned parameters^22,51^. Nevertheless, there is a consensus concerning the improvement of learning and memory induced by EE exposure^1,22^. In line with this, we show here that expression of bidirectional STDP within a short temporal window is favoured by exposure to EE.

*Ex vivo* long-term plasticity at excitatory synapses is generally examined with pharmacological blockade of GABA_A_R-mediated transmission to isolate glutamatergic transmission. We previously showed in striatum that GABA controls polarity of STDP^16,17^. Indeed, Hebbian and anti-Hebbian polarities of cortico-striatal STDP have been observed^41^ depending on the use (Hebbian STDP^38,39^) or not (anti-Hebbian STDP^34,37,40^) of GABA_A_R antagonists. This is in agreement with *in vivo* experiments, in which GABA transmission was not blocked (although it should be noted that anaesthesia affects GABAergic tone), showing an anti-Hebbian cortico-striatal STDP^42^ (and the present study). However, corticostriatal Hebbian STDP was elicited with pharmacological manipulation of GABAergic, dopaminergic and adenosine transmission^47^. In these *in vivo* corticostriatal STDP studies^42,47^, the recording duration of MSNs was between 10 and 20 minutes, which remains relatively short for STDP estimation. Indeed, STDP full expression does not generally occur during the first 15 minutes after pairings, and the initial fluctuations of synaptic weights do not necessarily predict long-term plasticity after one-hour recording. Nevertheless, it remains to be determined which STDP-like mechanisms are involved in awake and behaving rodents. It has been proposed that anti-Hebbian STDP would disfavour predictable inputs, keep synapses weak, and would allow novel sensory inputs to be better represented^14,52^. Therefore, anti-Hebbian STDP could be a crucial requirement for striatum, which acts as a coincident detector of distributed patterns of cortical and thalamic activity.

EE reduces inhibitory GABAergic transmission^53^ and restores impaired hippocampus-based memory tasks in a mouse model of Down syndrome^54^. Thus, EE constitutes a non-invasive and non-pharmacological way for reduction of the GABAergic signalling. It remains to be determined whether the tonic and phasic GABAergic components are equally affected by EE exposure. Indeed, developmental control of corticostriatal STDP and expression of anti-Hebbian bidirectional STDP depend mainly on tonic GABAergic signalling^17^. In addition, in the barrel cortex, in *in vivo* anaesthetised rats, tLTP occurrence depends on GABAergic tone^18^. The temporal (Δt_STDP_) shift of the occurrence of striatal tLTP on the post-pre pairing side depending on the SE/EE and/or anaesthetic regime suggests that the plasticity machinery is present but tLTP cannot be expressed. One hypothesis, in line with Gambino and Holtmaat (2012), is that GABAergic circuits are activated or inactivated depending on the Δt_STDP_ and environment/anaesthesia regime, thus precluding or favouring, respectively, the occurrence of tLTP. Therefore, by decreasing the inhibitory tone^53^, EE would promote tLTP expression, whereas anaesthesia would disfavour tLTP.

The effects of EE on plasticity, and in particular on LTP, have been mainly tested *ex vivo* (acute brain slices) in the neocortex or the hippocampus and remain controversial^51^. This is probably due to the wide variety of EE conditions (for example age, sex and strain differences, amount of enrichment or duration of EE exposure) used across studies^7^. Effects of EE on hippocampal neurogenesis, neuronal morphology and synaptic plasticity vary as a function of the duration of EE and the age of initial exposure to EE^55^. This is also exemplified in MSNs of the dorsal striatum, since 2–3 months of EE produced higher spine densities^56^ and higher accumulation of delta-fosB^57^ but no noticeable change was detected, in terms of cell volume and dendritic length, after 4–5 months of EE exposure^58^. We exposed rats to EE for eight weeks after weaning, and it has been shown that this EE condition promotes LTP (with a rate-coding paradigm) and an increase in spine density in the hippocampus^21,55,59^; note that six weeks EE was not sufficient to promote significant hippocampal LTP. In a mouse model of Fragile X syndrome, in which the tLTP induction threshold was increased in the prefrontal cortex, tLTP was restored to wild-type levels after exposure to EE for one month^60^.

Various neuromodulators (such as dopamine, noradrenaline, acetylcholine, brain-derived neurotrophic factor (BDNF), NO, GABA) control STDP expression and polarity (for reviews see^20,61^). In the striatum, dopamine is required for STDP expression *in vitro*^38,39,62^ and *in vivo*^42,47,63^ and has been shown to transform eligibility traces into plasticity^63^; BDNF promotes endocannabinoid-mediated STDP^64^ and GABA acts as an anti-Hebbian/Hebbian switch for STDP polarity^16,17,37^. Effects of neuromodulators on STDP have been extensively investigated in the hippocampus and prefrontal cortex^2,4,51^, but remain less documented in the striatum. Striatal dopamine level seems to be inert to EE or even slightly decreased^51^. Therefore, dopamine should not impact significantly STDP expression in SE vs EE. In the somatosensory cortex, 4–6 weeks of EE exposure induced a functional and structural plasticity by sharpening cortical whisker representation *in vivo* in adult rats^65,66^ and resulted in BDNF increase^4,67^. If a similar effect occurs in the striatum, endocannabinoid-mediated tLTD and tLTP^64^ and NMDAR-mediated LTP^68^ would be then favoured (because these forms of plasticity are controlled by BDNF), and less failure in STDP expression should be observed. This is in line with our observations, although additional data are required to firmly determine the STDP expression map in EE. EE, by decreasing GABAergic inhibition, as shown in the cerebral cortex^53^, is expected to stabilize an anti-Hebbian striatal STDP, as reported in our study (Fig. 6).

Exposure to EE has been tested in rodent models of various nervous system disorders, mainly in pathology for which no pharmacological treatments are available (such as Huntington’s and Alzheimer diseases or Rett’s and Down’s syndrome), and beneficial effects have been reported^2,4^. EE can also serve as a non-pharmacological treatment for cognitive enhancement^3,69–71^. Our study shows the crucial impact of environment on *in vivo* plasticity at the single-cell level in an associative Hebbian synaptic plasticity such as STDP. This stresses the fact that together with *in vivo* recordings in awake animals (patch-clamp, two-photon or multi-channel recordings), it is crucial to perform such challenging experiments in animals subjected to EE exposure^7^. EE exposure would guarantee normal levels of plasticity compared to SE, this later would mask all the benefit derived from *in vivo* recordings. Although it is tempting to parallel EE exposure in rodents and the cognitive reserve theory in humans^72^, it would be essential to estimate at the single-cell level the precise change of associative memory and learning in awake and behaving animals subjected to various forms of EE.

## Supplementary information

Supplementary Figures and legends

## Data Availability

All experimental data are available on request.

## References

[1] Van Praag H, Kempermann G, Gage FH (2000). Neural consequences of environmental enrichment. Nat Rev Neurosci.

[2] Nithianantharajah J, Hannan AJ (2006). Enriched environments, experience-dependent plasticity and disorders of the nervous system. Nat rev Neurosci.

[3] Sale A, Berardi N, Maffei L (2009). Enrich the environment to empower the brain. Trends Neurosci.

[4] Baroncelli L (2010). Nurturing brain plasticity: impact of environmental enrichment. Cell Death Differ.

[5] Ruediger S (2011). Learning-related feedforward inhibitory connectivity growth required for memory precision. Nature.

[6] Rosenweig MR, Bennett EL, Hebert M, Morimoto H (1978). Social grouping cannot account for cerebral effects of enriched environment. Brain Research.

[7] Simpson J, Kelly JP (2011). The impact of environmental enrichment in laboratory rats–behavioural and neurochemical aspects. Behav Brain Res..

[8] Martin SJ, Morris RGM (2002). New life in an old idea: The synaptic plasticity and memory hypothesis revisited. Hippocampus.

[9] Nabavi S (2014). Engineering a memory with LTD and LTP. Nature..

[10] Markram, H., Gerstner, W. & Sjöström, P.J. A history of spike-timing-dependent plasticity. *Front Synaptic Neurosci* 3, 4, 10.0.13.61/fnsyn.2011.00004 (2011). 10.3389/fnsyn.2011.00004 PMC3187646 22007168

[11] Feldman DE (2012). The spike-timing dependence of plasticity. Neuron.

[12] Froemke RC, Poo MM, Dan Y (2005). Spike-timing-dependent synaptic plasticity depends on dendritic location. Nature.

[13] Letzkus J, Kampa BM, Stuart GJ (2006). Learning rules for spike timing-dependent plasticity depend on dentritic synapse location. J Neurosci.

[14] Sjöström PJ, Häusser M (2006). A cooperative switch determines the sign of synaptic plasticity in distal dendrites of neocortical pyramidal neurons. Neuron.

[15] Froemke RC, Letzkus JJ, Kampa BM, Hang GB, Stuart GJ (2010). Dendritic synapse location and neocortical spike-timing-dependent plasticity. Front Synaptic Neurosci.

[16] Paillé V (2013). GABAergic circuits control spike-timing-dependent plasticity. J Neurosci.

[17] Valtcheva S (2017). Developmental control of spike-timing-dependent plasticity by tonic GABAergic signaling in striatum. Neuropharmacology..

[18] Gambino F, Holtmaat A (2012). Spike-timing-dependent potentiation of sensory surround in the somatosensory cortex is facilitated by deprivation-mediated disinhibition. Neuron.

[19] Pawlak V, Wickens JR, Kirkwood A, Kerr JN (2010). Timing is not everything: neuromodulation opens the STDP gate. Front Synaptic Neurosci.

[20] Foncelle A (2018). Modulation of Spike-Timing Dependent Plasticity: Towards the Inclusion of a Third Factor in Computational Models. Front Comput Neurosci..

[21] Alvarez VA, Sabatini BL (2007). Anatomical and physiological plasticity of dendritic spines. Annu Rev Neurosci.

[22] Eckert MJ, Abraham WC (2013). Effects of environmental enrichment exposure on synaptic transmission and plasticity in the hippocampus. Curr Top Behav Neurosci.

[23] Eckert MJ, Abraham WC (2010). Physiological effects of enriched environment exposure and LTP induction in the hippocampus *in vivo* do not transfer faithfully to *in vitro* slices. Learn Mem.

[24] Madronal N (2010). Effects of enriched physical and social environments on motor performance, associative learning, and hippocampal neurogenesis in mice. PLoS One.

[25] Yin HH (2009). Dynamic reorganization of striatal circuits during the acquisition and consolidation of a skill. Nat Neurosci..

[26] Koralek AC, Jin X, Long JD, Costa RM, Carmena JM (2012). Corticostriatal plasticity is necessary for learning intentional neuroprosthetic skills. Nature.

[27] Shan, Q., Ge, M., Christie, M.J. & Balleine, B.W. The acquisition of goal-directed actions generates opposing plasticity in direct and indirect pathways in dorsomedial striatum. *J Neurosci*. **34**, 9196–9201, %2010.1523/JNEUROSCI.0313-14.2014 (2014). 10.1523/JNEUROSCI.0313-14.2014 PMC6608360 25009253

[28] Hawes SL (2015). Multimodal Plasticity in Dorsal Striatum While Learning a Lateralized Navigation Task. J Neurosci.

[29] Rothwell PE (2015). Input- and Output-Specific Regulation of Serial Order Performance by Corticostriatal Circuits. Neuron.

[30] Xiong Q, Znamenskiy P, Zador AM (2015). Selective corticostriatal plasticity during acquisition of an auditory discrimination task. Nature..

[31] Hannan AJ (2014). Environmental enrichment and brain repair: harnessing the therapeutic effects of cognitive stimulation and physical activity to enhance experience-dependent plasticity. Neuropathol Appl Neurobiol.

[32] Wilson CJ, Kawaguchi Y (1996). The origins of two-state spontaneous membrane potential fluctuations of neostriatal spiny neurons. J Neurosc.

[33] Pidoux M, Mahon S, Deniau JM, Charpier S (2011). Integration and propagation of somatosensory responses in the corticostriatal pathway: an intracellular study *in vivo*. J Physiol..

[34] Fino E, Glowinski J, Venance L (2005). Bidirectional activity-dependent plasticity at corticostriatal synapses. J Neurosci.

[35] Mahon S, Delord B, Deniau JM, Charpier S (2000). Intrinsic properties of rat striatal output neurons and time-dependent facilitation of cortical inputs *in vivo*. J Physiol.

[36] Buchanan KA, Mellor JR (2010). The activity requirements for spike timing-dependent plasticity in the hippocampus. Front Synaptic Neurosci..

[37] Fino E (2010). Distinct coincidence detectors govern the corticostriatal spike timing-dependent plasticity. J Physiol.

[38] Pawlak V, Kerr JN (2008). Dopamine receptor activation is required for corticostriatal spike-timing-dependent plasticity. J Neurosci.

[39] Shen W, Flajolet M, Greengard P, Surmeier DJ (2008). Dichotomous dopaminergic control of striatal synaptic plasticity. Science.

[40] Valtcheva S, Venance L (2016). Astrocytes gate Hebbian synaptic plasticity in the striatum. Nat Commun.

[41] Fino E, Venance L (2010). Spike-timing dependent plasticity in the striatum. Front Synaptic Neurosci.

[42] Schulz JM, Redgrave P, Reynolds JN (2010). Cortico-striatal spike-timing dependent plasticity after activation of subcortical pathways. Front Synaptic Neurosc.

[43] Mahon S, Deniau JM, Charpier S (2001). Relationship between EEG potentials and intracellular activity of striatal and cortico-striatl neurons: an *in vivo* study under different anesthetics. Cereb Cortex.

[44] Taub AH, Katz Y, Lampl I (2013). Cortical balance of excitation and inhibition is regulated by the rate of synaptic activity. J Neurosci.

[45] Lovinger DM, Zimmerman SA, Levitin M, Jones MV, Harrison NL (1993). Trichloroethanol potentiates synaptic transmission mediated by gamma-aminobutyric acid_A_ receptors in hippocampal neurons. J Pharmacol Exp Ther.

[46] Perrin E, Venance L (2019). Bridging the gap between striatal plasticity and learning. Curr Opin Neurobiol..

[47] Fisher SD (2017). Reinforcement determines the timing dependence of corticostriatal synaptic plasticity *in vivo*. Nat Commun.

[48] Planert H, Berger TK, Silberberg G (2013). Membrane properties of striatal direct and indirect pathway neurons in mouse and rat slices and their modulation by dopamine. PLoS ONE.

[49] Day M, Wokosin D, Plotkin JL, Tian X, Surmeier DJ (2008). Differential excitability and modulation of striatal medium spiny neuron dendrites. J Neurosci..

[50] Cui Y, Prokin I, Mendes A, Berry H, Venance L (2018). Robustness of STDP to spike timing jitter. Sci Rep..

[51] Hirase H, Shinohara Y (2014). Transformation of cortical and hippocampal neural circuit by environmental enrichment. Neuroscience.

[52] Roberts PD, Bell CC (2000). Computational consequences of temporally asymmetric learning rules: II. Sensory image cancellation. J Comput Neurosci..

[53] Sale A (2007). Environmental enrichment in adulthood promotes amblyopia recovery through a reduction of intracortical inhibition. Nat Neurosci..

[54] Begenisic T (2011). Environmental enrichment decreases GABAergic inhibition and improves cognitive abilities, synaptic plasticity, and visual functions in a mouse model of Down syndrome. Front Cell Neurosci.

[55] Hosseiny S (2015). Differential neuronal plasticity in mouse hippocampus associated with various periods of enriched environment during postnatal development. Brain Struct Funct..

[56] Turner CA, Lewis MH, King MA (2003). Environmental enrichment: effects on stereotyped behavior and dendritic morphology. Dev Psychobiol.

[57] Lafragette A, Bardo MT, Lardeux V, Solinas M, Thiriet N (2017). Reduction of cocaine-induced locomotor effects by enriched environment is associated with cell-specific accumulation of delta-FosB in striatal and cortical subregions. Int J Neuropsychopharmacol.

[58] Faherty CJ, Kerley D, Smeyne RJ (2003). A Golgi-Cox morphological analysis of neuronal changes induced by environmental enrichment. Brain Res Dev Brain Res.

[59] Duffy SN, Craddock KJ, Abel T, Nguyen PV (2001). Environmental enrichment modifies the PKA-dependence of hippocampal LTP and improves hippocampus-dependent memory. Learn Mem.

[60] Meredith RM, Holmgren CD, Weidum M, Burnashev N, Mansvelder HD (2007). Increased threshold for spike-timing-dependent plasticity is caused by unreliable calcium signaling in mice lacking fragile X gene FMR1. Neuron.

[61] Brzosko Z, Mierau SB, Paulsen O (2019). Neuromodulation of Spike-Timing-Dependent Plasticity: Past, Present, and Future. Neuron.

[62] Xu H (2018). Dopamine-endocannabinoid interactions mediate spike-timing-dependent potentiation in the striatum. Nat Commun.

[63] Yagishita S (2014). A critical time window for dopamine actions on the structural plasticity of dendritic spines. Science.

[64] Gangarossa G. *et al*. BDNF Controls Bidirectional Endocannabinoid Plasticity at Corticostriatal Synapses. *Cereb Cortex* **25**, 10.1093/cercor/bhz081 (2019). 10.1093/cercor/bhz081 31329835

[65] Polley DB, Kvasnák E, Frostig RD (2004). Naturalistic experience transforms sensory maps in the adult cortex of caged animals. Nature.

[66] Feldman DE, Brecht M (2005). Map plasticity in somatosensory cortex. Science.

[67] Gomez-Pinilla F, Ying Z, Agoncillo T, Frostig R (2011). The influence of naturalistic experience on plasticity markers in somatosensory cortex and hippocampus: effects of whisker use. Brain Research.

[68] Jia Y, Gall CM, Lynch G (2010). Presynaptic BDNF promotes postsynaptic long-term potentiation in the dorsal striatum. J Neurosci.

[69] Pang TYC, Hannan AJ (2013). Enhancement of cognitive function in models of brain disease through environment enrichment and physical activity. Neuropharmacology.

[70] Dresler M (2013). Non-pharmacological cognitive enhancement. Neuropharmacology.

[71] Kelly AM (2015). Non-pharmacological approaches to cognitive enhancement. Handbook of Experimental Pharmacology.

[72] Petrosini L (2009). On whether the environmental enrichment may provide cognitive and brain reserves. Brain Research Reviews.

