# Supplementary material for: Environmental enrichment shapes striatal spike-timing-dependent plasticity in vivo
Source: Sci Rep. 2019 Dec 19;9:19451. doi: 10.1038/s41598-019-55842-z (PMC6923403; doi:10.1038/s41598-019-55842-z)
Supplement: Supplementary file 1 — Supplementary Figures and legends [file 41598_2019_55842_MOESM1_ESM.pdf]

Environmental enrichment shapes striatal spike-timing-dependent plasticity *in vivo*

Teresa MORERA-HERRERAS, Yves GIOANNI, Sylvie PEREZ, Gaetan VIGNOUD and

Laurent VENANCE

SUPPORTING INFORMATION: Supplementary Figures

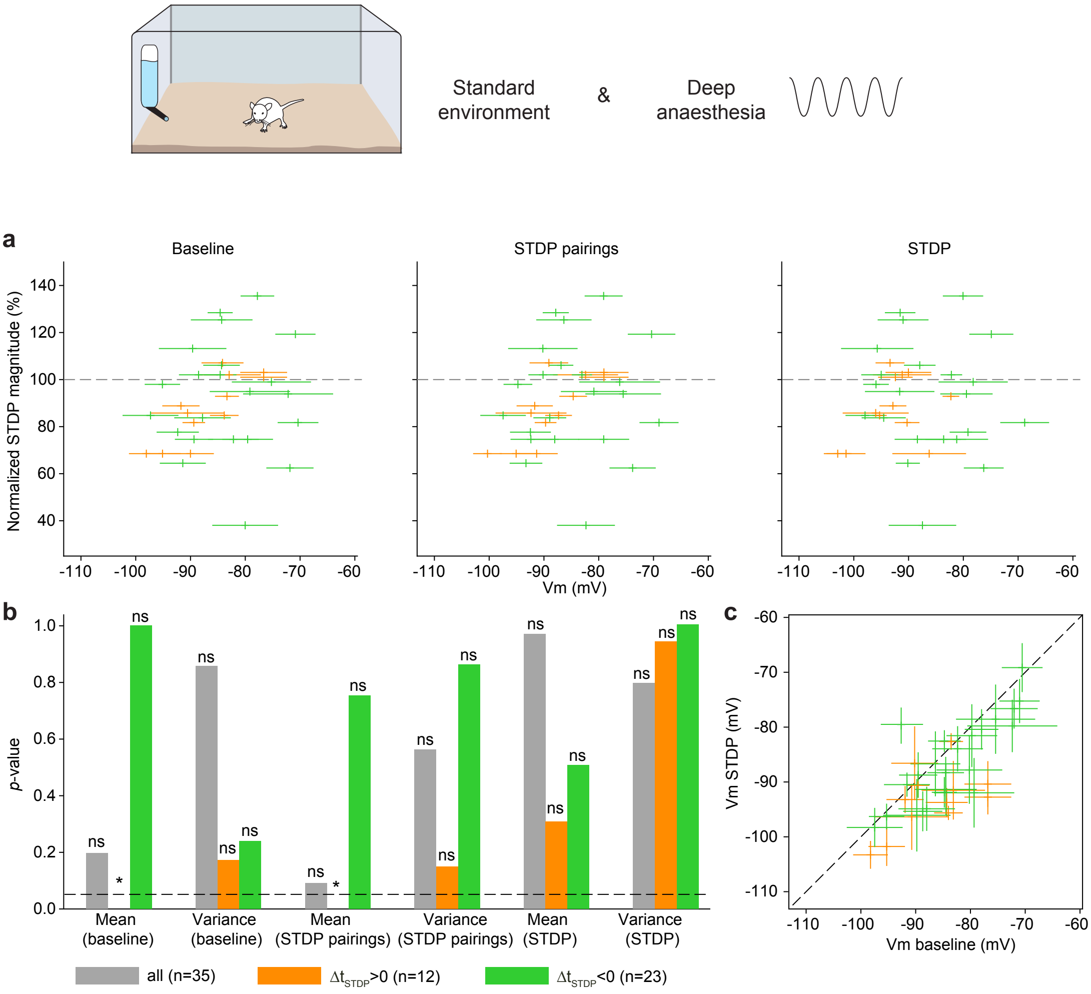

Supplementary Figure 1. Vm analysis during *in vivo* STDP in deeply anaesthetised SE rats (related the main Figure 2)

(**A**) Relative changes in EPSP amplitude as a function of the mean Vm before (baseline) (left), during (middle) and after (the last 5 minutes of recordings) (right) STDP pairings. (**B**) *P*-value of the Spearman correlation test, between the relative change in EPSP amplitude and the mean

or variance of Vm before stimulation (baseline), during and after (the last 5 minutes of recordings) STDP pairings. These correlations where established either considering all STDP experiments (grey), only pre-post pairings (orange) and post-pre pairings (green). These values are reported in the histogram plot (with dotted line representing a $p=0.05$ and * $p<0.05$). (**C**) Mean Vm after STDP pairings (the last 5 minutes of recordings) as a function of mean Vm before stimulation during baseline. Pearson correlation test coefficient: 0.742 and $p<0.05$.
Pre-post and post-pre pairings are illustrated in orange and green, respectively. See Methods for detailed information on Vm computation; Vm was measured just before each cortical stimulation or pairing. Error bars represent the SD.

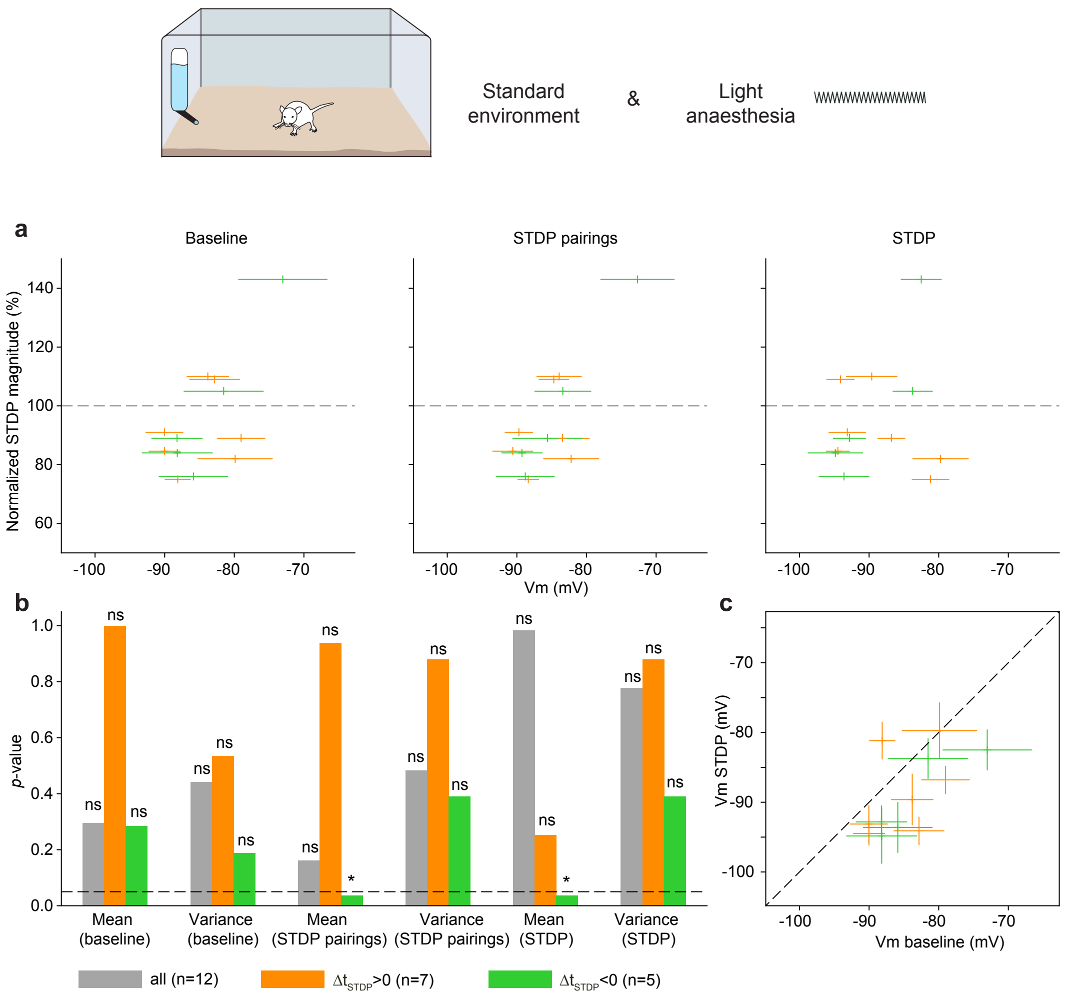

Supplementary Figure 2. Vm analysis during *in vivo* STDP in light anaesthetised SE rats (related the main Figure 4)

(**A**) Relative changes in EPSP amplitude as a function of the mean Vm before (baseline) (left), during (middle) and after (the last 5 minutes of recordings) (right) STDP pairings. (**B**) *P*-value of the Spearman correlation test, between the relative change in EPSP amplitude and the mean or variance of Vm before stimulation (baseline), during and after (the last 5 minutes of recordings) STDP pairings. These correlations where established either considering all STDP experiments (grey), only pre-post pairings (orange) and post-pre pairings (green). These values are reported in the histogram plot (with dotted line representing a p=0.05 and * p<0.05). (**C**)

Mean Vm after STDP pairings (the last 5 minutes of recordings) as a function of mean Vm before stimulation during the baseline. Pearson correlation test coefficient: 0.615 and $p<0.05$. Pre-post and post-pre pairings are illustrated in orange and green, respectively. See Methods for detailed information on Vm computation; Vm was measured just before each cortical stimulation or pairing. Error bars represent the SD.

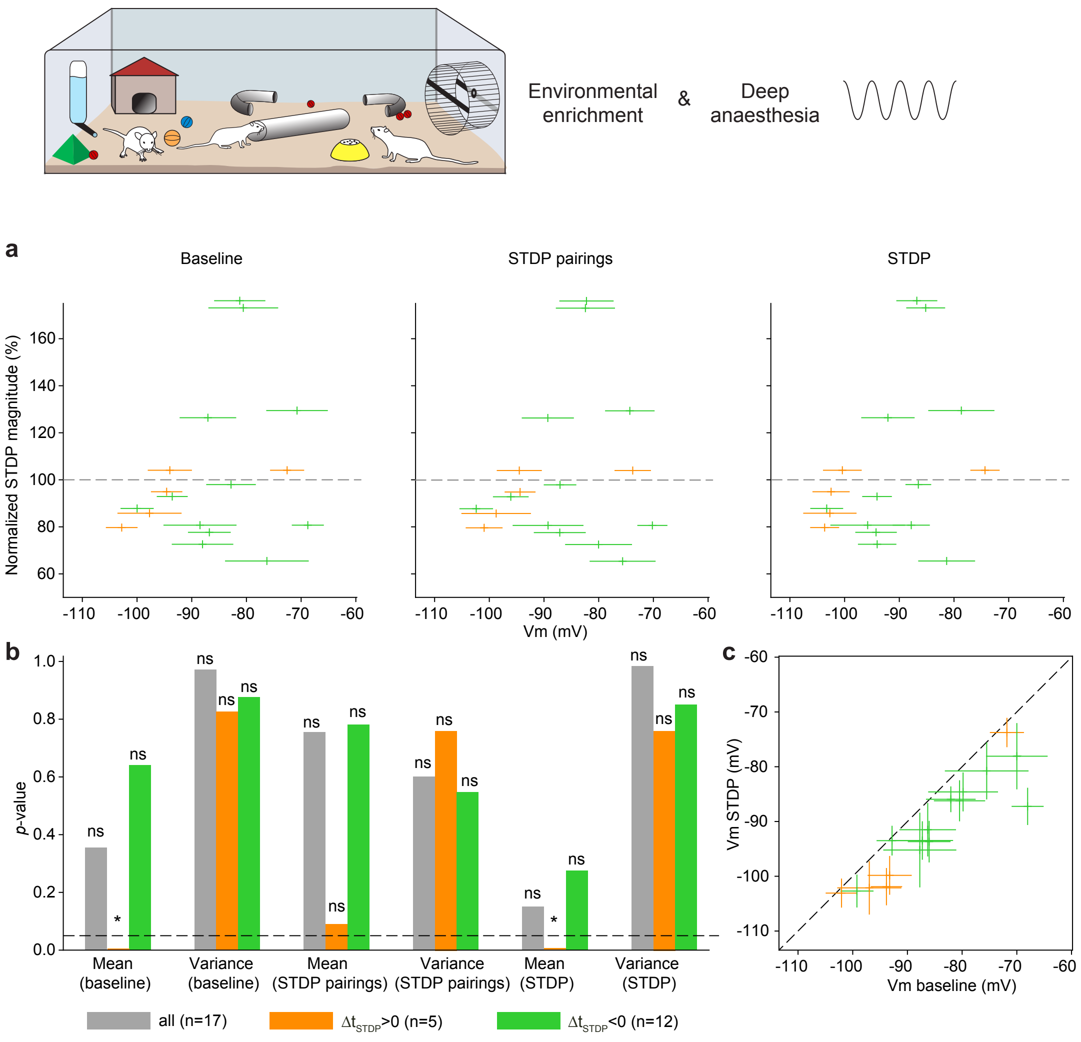

Supplementary Figure 3. Vm analysis during *in vivo* STDP in deeply anaesthetised EE rats (related the main Figure 5)

(**A**) Relative changes in EPSP amplitude as a function of the mean Vm before (baseline) (left), during (middle) and after (the last 5 minutes of recordings) (right) STDP pairings. (**B**) *P*-value of the Spearman correlation test, between the relative change in EPSP amplitude and the mean or variance of Vm before stimulation (baseline), during and after (the last 5 minutes of recordings) STDP pairings. These correlations where established either considering all STDP experiments (grey), only pre-post pairings (orange) and post-pre pairings (green). These values are reported in the histogram plot (with dotted line representing a p=0.05 and * p<0.05). (**C**)

Mean Vm after STDP pairings (the last 5 minutes of recordings) as a function of mean Vm before stimulation during the baseline. Pearson correlation test coefficient: 0.917 and $p<0.05$. Pre-post and post-pre pairings are illustrated in orange and green, respectively. See Methods for detailed information on Vm computation; Vm was measured just before each cortical stimulation or pairing. Error bars represent the SD.

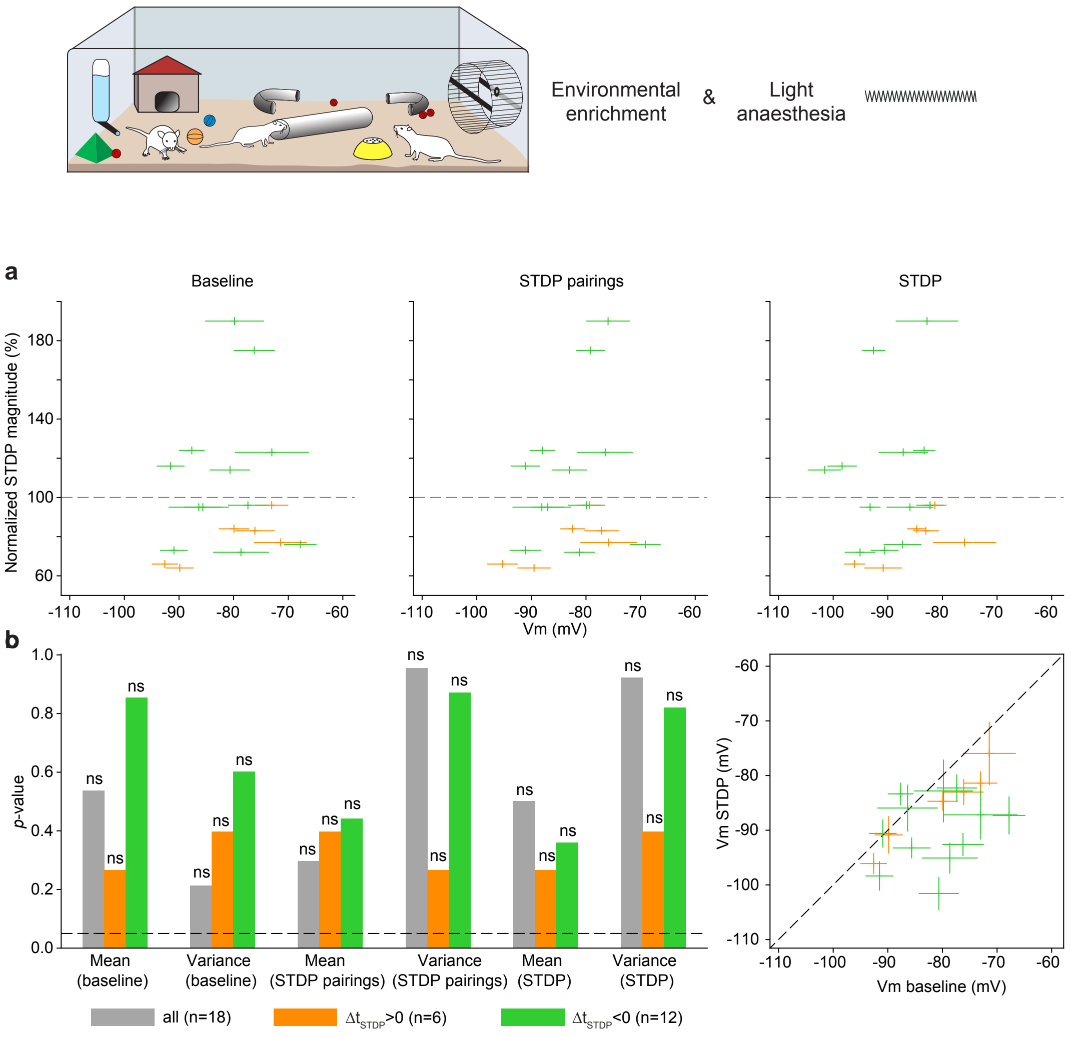

Supplementary Figure 4. Vm analysis during *in vivo* STDP in light anaesthetised EE rats (related the main Figure 6)

(**A**) Relative changes in EPSP amplitude as a function of the mean Vm before (baseline) (left), during (middle) and after (the last 5 minutes of recordings) (right) STDP pairings. (**B**) *P*-value of the Spearman correlation test, between the relative change in EPSP amplitude and the mean or variance of Vm before stimulation (baseline), during and after (the last 5 min of recording) STDP pairings. These correlations where established either considering all STDP experiments (grey), only pre-post pairings (orange) and post-pre pairings (green). These values are reported in the histogram plot (with dotted line representing a p=0.05 and * p<0.05). (**C**) Mean Vm after

STDP pairings (the last 5 minutes of recordings) as a function of mean Vm before stimulation during the baseline. Pearson correlation test coefficient: 0.494 and $p<0.05$.

Pre-post and post-pre pairings are illustrated in orange and green, respectively. See Methods for detailed information on Vm computation; Vm was measured just before each cortical stimulation or pairing. Error bars represent the SD.
